# Upscaling, toxicity and efficacy of multifaceted dressing embedded with dsirna-loaded gold nanoparticles for enhancing diabetic wound treatment

**DOI:** 10.1371/journal.pone.0327375

**Published:** 2025-09-05

**Authors:** Farha Yasmin Faris Taufeq, Muhammad Luqman Nordin, Haliza Katas

**Affiliations:** 1 Faculty of Pharmacy, Centre for Drug Delivery Technology and Vaccine (CENTRIC), Universiti Kebangsaan Malaysia, Jalan Raja Muda Abdul Aziz, Kuala Lumpur, Malaysia,; 2 Department of Pharmaceutical Technology, Faculty of Pharmacy, University Malaya, Kuala Lumpur, Malaysia; Chapman University, UNITED STATES OF AMERICA

## Abstract

Poor vascularization and infections hinder diabetic wound healing, posing challenges in therapy development. A multi-action approach incorporating Dicer-substrate small interfering RNA (DsiRNA) against the prostaglandin transporter (PGT) gene and gold nanoparticles (AuNPs) into a Pluronic F-127 (PF127) gel was developed. This study aimed to upscale AuNP biosynthesis using *Lignosus rhinocerotis* (tiger milk mushroom, TMM) extract and chitosan as stabilizers. Response Surface Methodology (RSM) optimized the synthesis with 0.6 mL chloroauric acid (HAuCl₄) and 4.4 mL TMM extract, producing upscaled AuNPs (152.93 ± 1.56 nm, + 30 mV) with a Surface Plasmon Resonance peak at 538 nm. Both lab-scale and upscaled AuNPs exhibited antibacterial activity against *Staphylococcus aureus* and *Pseudomonas aeruginosa* (MIC: 250 μg/mL). The gel formulation demonstrated favourable gelling properties for wound dressing. A 28-day toxicity study confirmed no adverse effects on haematology, biochemistry, or organ morphology. In diabetic Wistar rats, only the wounds treated with Pluronic gels containing AuNPs-DsiRNA showed no signs of infection, while the other groups exhibited infection and pustules in the wounded areas. These findings highlight the potential of AuNP-DsiRNA thermoresponsive gels as an innovative, safe, and effective therapy for diabetic wound healing.

## 1. Introduction

The prevalence and incidence of diabetes is increasing, with increasing morbidity and mortality rates and premature death. Based on data reported in the International Diabetes Federation (IDF) Tenth Edition 2021, it is estimated that approximately 537 million adults have diabetes, of which 541 million adults are at increased risk of developing Type 2 Diabetes. The sharp increase in the number is expected to reach 783 million by 2045 [1]. Diabetic ulcers, one of the implications of this disease, have become a major economic burden and can affect a person’s productivity and quality of life [2].

Diabetic foot ulcers (DFUs) are a common and serious complication of diabetes, affecting 10–25% of people with the disease [1]. These ulcers arise from a combination of factors, including sensory neuropathy, changes in foot anatomy, and repetitive stress from walking. Sensory neuropathy leads to a loss of sensation in the feet, making it difficult for patients to detect and respond to minor injuries, while daily activities can exacerbate trauma. Additional factors such as reduced blood flow and oedema further contribute to the development and worsening of DFUs. Left untreated, these wounds often remain open, posing a significant risk of infection and amputation. Once ischemic symptoms appear, aggressive treatment becomes critical to minimizing the risk of lower limb amputation [3,4].

Impaired healing makes DFUs highly susceptible to infections, with approximately 50% of non-healing ulcers becoming infected due to weakened immunity. Bacterial biofilms frequently develop in these wounds, further worsening the prognosis [5]. Infected DFUs not only increase the risk of amputation but also contribute to higher mortality rates [6]. The rise of multidrug-resistant infections exacerbates these challenges, emphasizing the urgent need for innovative and effective wound care therapies beyond traditional antibacterial approaches.

Previous studies have demonstrated the efficacy of gold nanoparticles (AuNPs) in treating diabetic wounds, primarily due to their lower toxicity compared to silver nanoparticles (AgNPs). In this study, AuNPs were synthesized using a green synthesis method that employed Tiger Milk Mushroom (TMM) extract and chitosan as reducing and stabilizing agents, respectively. Stabilizing agents such as chitosan play a crucial role in preventing agglomeration, maintaining uniform nanoparticle size, and imparting a positive charge to the particles. Uncoated AuNPs, on the other hand, are highly sensitive to environmental factors like pH, temperature, and electrolytes, making them prone to aggregation. To ensure colloidal stability, protective species containing thiol or amine groups are often incorporated during synthesis [7]. Chitosan, a biocompatible polysaccharide and mucoadhesive polymer, further enhances nanoparticle diffusion and absorption [8], making it an ideal stabilizer for ensuring the stability and efficacy of AuNPs in treating diabetic wound infections. Given their promising biomedical applications, efforts have been made to upscale AuNP production for commercial and clinical use.

Upscaling AuNPs has gained industry attention due to its ease of preparation, physicochemical stability, and scalability, especially through green synthesis, which minimizes environmental impact [9]. Biosynthesis using microorganisms like bacteria and fungi is popular for its eco-friendliness, lower cost, biocompatibility, and low toxicity [10,11]. However, large-scale production introduces challenges such as maintaining particle size uniformity, ensuring process reproducibility, and optimizing synthesis parameters to comply with regulatory standards [12,13]. To address these challenges, mathematical modelling plays a crucial role in optimizing scale-up production by aiding in process control, reducing waste, and improving efficiency [14,15]. Meanwhile, biological synthesis provides a sustainable alternative to chemical methods by utilizing natural stabilizers, reducing the risk of aggregation and contamination. This integrated approach enhances the feasibility of large-scale AuNP manufacturing, which is essential for their commercialization and clinical applications [16].

After synthesis, the AuNPs are adsorbed with DsiRNA, a gene silencing agent, and incorporated into a thermoresponsive Pluronic PF127 gel. The DsiRNA component in this formulation specifically targets and silences genes that are overexpressed in diabetic wounds, such as the prostaglandin transporter (PGT) gene, which is closely related to slow wound healing. By reducing inflammation and promoting tissue repair, the DsiRNA component plays a crucial role in the healing process. This gel solidifies at physiological temperatures (25–37°C) depending on PF127 concentration, making it an excellent candidate for diabetic wound treatment with different sizes [17,18]. Recent research highlights the benefits of incorporating AuNPs-DsiRNA into PF127 and PEG 400-based thermoresponsive gels, which exhibit good spreadability, adhesion, and non-toxicity to human dermal fibroblasts [19]. These nanocomposite gels not only promote enhanced cell migration but also demonstrate significant wound healing potential in diabetic mouse models of Type 1 Diabetes [20]. Scaling up nanosized-based drug delivery systems presents significant challenges in the development of nanomedicine for wound delivery. The findings of this study will provide valuable insights into the feasibility of translating this innovation into clinical applications. Building on this evidence, the current study investigates the gel’s toxicity, wound-healing efficacy, and scalability to ensure its feasibility as a therapeutic solution for diabetic wounds.

## 2. Materials and methods

### 2.1. Materials

Gold (III) chloride hydrate (HAuCl₄) (99.995% trace metals basis) was sourced from Sigma-Aldrich (Malaysia) as the gold precursor for synthesizing AuNPs. *Lignosus rhinocerotis* sclerotial powder, used as a green reducing agent, was generously provided by LignasBio Synergy Plt., Selangor, Malaysia. Low-molecular-weight chitosan (LMW-CS) (190 kDa, 75–85% degree of deacetylation), serving as the stabilizing agent, and Pluronic PF-127 powder for gel formulation were obtained from Sigma-Aldrich (Ireland and Malaysia, respectively). For antibacterial study, Mueller Hinton Agar and Mueller Hinton Broth was obtained from Thermo Scientific^TM^ Oxoid^TM^. 2,3,5-triphenyltetrazolium chloride (TTC) were supplied by Sigma -Aldrich (USA). DsiRNA targeting the PGT gene [27 bp, sequences: 5′-rGrArArGrGrArArGrUrGrGrCrUrGrArGrUrUrArUrUrArATA-3′ (sense strand) and 5’-rUrArUrUrArArUrUrArCrUrCrArGrCrCrArCrUrUrCrCrUrUrCrUrU-3’ (antisense strand)] was purchased from Integrated DNA Technologies (IDT, USA). For gel formulation, glacial acetic acid (99.7% purity) was sourced from R&M Chemicals, UK, and polyethylene glycol 400 (PEG 400, molecular weight 380–420 g/mol) was procured from Merck (Darmstadt, Germany). Milli-Q water (Elga Purelab, USA) and distilled water (prepared using a Hamilton WCS/85 Cabinet Water Still) were used throughout the study.

Subacute toxicity studies utilized isoflurane (Piramal Critical Care Inc., India), Intrasite Gel (Smith & Nephew, UK), Tegaderm (3M, USA), EDTA blood collection tubes, serum collection tubes (VACUTAINER®, USA), 10% buffered formalin, and hematoxylin and eosin (H&E) for tissue analysis. In vivo wound healing studies employed Wistar rats and anesthetic agents (Ketamine, Xylazine, and Tiletamine [KTX], 0.1 mL/100 g), as approved by the Animal Ethics Committee of Universiti Kebangsaan Malaysia (UKM). Streptozotocin (STZ, U-9889), used to induce diabetes in the rat model, was obtained from Santa Cruz Biotechnology, USA. While for the gene expression analysis, RNA Protect Tissue Reagent, RNeasy Fibrous Tissue Mini Kit, Quantitect Reverse Transcription Kit, and QuantiNova SYBR Green PCR Kit were supplied by Qiagen, Germany.

### 2.2. Methods

#### 2.2.1. Preparation of TMM extract.

TMM sclerotium powder was extracted by hot water extraction method [19]. The sclerotium powder was boiled in distilled water at a ratio of 1:20 (w/v) at 90–95 °C for 60 minutes. Then, the mixture was filtered using Whatman No.1 filter paper and the obtained residue was re-extracted for a second time. Then, the extract solution was centrifuged at 10 000 rpm for 30 minutes and frozen at −110°C. The resulting extract was stored at −20°C before being used as a reducing agent for the synthesis of AuNPs.

#### 2.2.2 Experimental design.

RSM was used to optimize the scale-up of AuNPs using Design-Expert® software (trial version 13; Stat-Ease Inc., Minneapolis, MN, USA). Central composite design (CCD) was used as an optimization tool to evaluate and optimize the effects of two independent variables, namely the concentration of HauCl_4_ and TMM extract. Meanwhile, the absorbance wavelength of AuNPs (to characterize the reaction product) was set as the dependent variable. Before conducting RSM, preliminary studies of AuNPs synthesis were carried out to determine the appropriate concentrations of HAuCl_4_ and TMM extract.

#### 2.2.3. Biologically Large-Scale Synthesis of AuNPs.

AuNPs were synthesized according to a previously reported method with minor modifications [19]. First, a 1% w/v chitosan solution was prepared by dissolving 10 g chitosan in 1000 mL 1% v/v acetic acid under magnetic stirring at 500 rpm for 12 h at 40–50°C. Based on RSM, lab scale and up-scaled AuNPs were prepared as follows: 4.4 mL 0.125 mg/mL TMM extract and 0.6 mL 0.1 M HAuCl4 solution were added to the chitosan solution and stirred for 10 min. The mixture was then sonicated using a Branson Ultrasonic Cleaner, Model 3510, for 3–5 h until a wine-purple colour was observed. Scale-up was performed using a 100-fold increase in volume from the lab scale. Centrifugation at 10,000 rpm was performed for 30 minutes to collect the AuNPs pellet, the supernatant was then removed and replaced with distilled water. Pure AuNPs were stored at −80°C before freeze-drying at −110°C. The freeze-dried AuNPs were stored at −20 °C for further analysis.

#### 2.2.4. Characterization for lab scale and up-scaled AuNPs.

The formation of lab scale and up-scaled AuNPs was determined using a Shimadzu 1800 UV-Vis spectrophotometer (Japan), with the scanning range set from 300 to 600 nm at a moderate speed, and baseline correction performed using a blank reference.

Fourier transform infrared spectroscopy (FTIR) was carried out to identify the functional group present in the synthesized nanoparticles. Lyophilized TMM, chitosan, and AuNP were prepared for FTIR-ATR analysis, with spectra recorded using a Perkin Elmer 100 Spectrum (Waltham, MA, USA) in the range of 4000–400 cm ⁻ ¹, using 32 scans at a resolution of 4 cm ⁻ ¹.

The Z-average particle size, polydispersity index (PDI), and zeta potential of AuNPs were measured in triplicate using a Malvern Zetasizer (Worcestershire, UK) at 25°C and a detection angle of 90°.

Morphological analysis of AuNPs was performed using a Philips CM12 TEM (USA). Nanoparticles were frozen at −80°C for three days in a Lexicon II ULT freezer, lyophilized using a ScanVac CoolSafe freeze-dryer. Then, the dried AuNPs powder was stirred in distilled water until evenly dispersed. For analysis, a droplet of sample dispersion was placed on a copper grid coated with amorphous carbon film and allowed to dry at room temperature for 1 min. The samples were observed using TEM at 260x and 600kx magnification under an operating voltage of 120 kV, then analyzed using Velox software [19].

#### 2.2.5. DsiRNA loading.

An equal volume of DsiRNA (15 μg/mL) was mixed into 1 mL of AuNPs suspension (0.25 mg/mL) for lab scale. While for the scale-up, 10 mL of DsiRNA (15 μg/mL) was mixed into 10 mL of AuNPs suspension (0.25 mg/mL). The resulting mixture was then incubated at room temperature for 30 minutes to allow adsorption of DsiRNA onto the AuNPs surface.

#### 2.2.6. Entrapment and binding efficiency.

Entrapment efficiency (EE) assay was determined using AuNPs-DsiRNA suspension that had been centrifuged for 30 minutes at 10 000 rpm (Empar Universal 320 R, Andreas Hettich GmbH & Co., Germany). The absorbance of the obtained supernatant was scanned using a UV spectrometer at 260 nm (Shimadzu 1800 High Resolution spectrophotometer (1 nm band width), Shimadzu, Kyoto, Japan). The EE of DsiRNA loaded onto AuNPs was calculated as follows:


$$EE\;DsiRNA,\;\%=\;\frac{\;(DsiRNA\;in\;sample-DsiRNA\;in\;supernatant)\;}{DsiRNA\;in\;sample}\;x\;100$$

The binding efficiency (BE) of DsiRNA adsorbed onto the AuNPs surface was determined using gel electrophoresis. The gel used was E-Gel® 4% agarose stained with ethidium bromide (Invitrogen, USA). Samples were prepared by adding the dye to each sample solution at a ratio of 1:5. Then, samples containing 20 μL of AuNPs-DsiRNA at different concentrations were added to the wells. Single DsiRNA was used as a positive control while a 10 bp (base pair) DNA ladder solution (Invitrogen, USA) was used as a size reference. Electrophoresis was run for 30 minutes and the DsiRNA migration bands were then observed under a UV transilluminator at 480 nm (Invitrogen, USA).

#### 2.2.7. Antibacterial tests.

***2.2.7.1.* *Inoculum Preparation by Growth Method***. *Staphylococcus aureus* (ATCC 25923) and *Pseudomonas aeruginosa* (ATCC 27853) were streaked on MHA plates and incubated at 37 °C overnight. Using a sterile loop, 3–5 colonies were transferred into 5 mL of MHB and incubated overnight for bacterial growth. The turbidity of the bacterial suspension was adjusted to 0.08–0.13 at 625 nm using a spectrophotometer to achieve a microbial count of 1 × 10⁸ CFU/mL.

***2.2.7.2. Microbroth dilution method***. The minimum inhibitory concentration (MIC) was determined via serial dilutions of AuNPs (starting at 2 mg/mL) in MHB. A bacterial suspension at 1 × 10⁸ CFU/mL was diluted to 1 × 10⁶ CFU/mL and further reduced to 5 × 10⁵ CFU/mL in 96-well plates containing AuNPs. After overnight incubation at 37 °C, 2,3,5-triphenyltetrazolium chloride (TTC) reagent was added as it is used as a colorimetric indicator and MIC values were recorded as the lowest concentration with no red colour and cloudy formation (no bacterial growth).

#### 2.2.8. Preparation of hydrogels.

Two types of hydrogels were prepared following a previously established method, with some modification (Nor Azlan et al. 2020): one was a blank Pluronic gel, and the other was a Pluronic gel incorporated with AuNPs-DsiRNA. The blank gel was made by dissolving 25% (w/v) PF127 in cold distilled water, then adding 22% (v/v) PEG and stirring the mixture at 4°C for 4 hours to produce 10 mL and 100 mL of gel for lab-scale and upscale respectively. The AuNPs-DsiRNA-loaded gel was prepared in a similar manner, with the addition of a solution containing 1.25 mg/mL AuNPs and 15 μg/mL DsiRNA (based on the optimal dose from antibacterial studies). This solution was mixed with the Pluronic gel containing PEG and stirred for an additional 2 hours at 4°C. The final gel was then characterized for its texture and physical appearance.

#### 2.2.9. Hydrogel characterization.

***2.2.9.1 Gelation Time*.** The gelation time (T_gel_) was determined by pouring 10 mL of the gel solution (4 °C) into a beaker containing a 3 cm magnetic stir bar and stirring at 200 rpm and heating to 100 °C. T_gel_ was determined when the magnetic stir bar stopped moving and this temperature was measured using a thermometer.

***2.2.9.2 Texture profile analysis (TPA).*** TPA was carried out using a texture analyzer (Brookfield CT-3, USA) operated in compression mode. The parameters were set as follows: target: 4.0 mm, trigger load at 0.1 N, test speed at 1 mm/s, dwell time 0 s and 2 number of cycles. Briefly, 10 mL of gel was placed in a glass jar. In each analysis, an analytical probe with a disc (diameter 36 mm) was inserted into a glass jar. TexturePro CT V1.5 Build 20 software was used to analyze the results and generate a graph of resultant force (N) versus time (s). The hardness, viscosity and cohesiveness of the gel were obtained from the plotted graphs.

***2.2.9.3 Rheology***. Rheometer (MCR 72, Anton Paar) TORC 5000 Operator was used to analyse the rheological profile of the gel formulated using a cone and plate geometry of 25 mm diameter and 2° angle at 25°C. The shear rate range was set from 0–500 s^-1^. Briefly, a small amount of sample was loaded onto the rheometer plate. The shear rate versus shear stress graph was plotted using Anton Paar Software: RheoCompass to determine the flow properties of the gel.

#### 2.2.10. *In Vitro* drug release of active agents.

In vitro drug release of AuNPs-DsiRNA was carried out by placing 5 mL of gel into a 20 mL beaker. Briefly, 5 mL of PBS (pH 7.4) was slowly added on top of the solidified gel. The gel was then placed in an incubator with the temperature maintained at 37 °C. A 1 mL sample was carefully taken every hour for 8 hours (1, 2, 3, 4, 5, 6, 7, 8), followed by every 24 hours (24 and 48 hours) to ensure that only the top layer of the sample was taken without disturbing the gel layer. The taken sample was replaced with 1 mL of PBS. Sample measurements were performed using a UV-vis spectrophotometer at wavelengths of 260 nm and 530 nm, respectively for DsiRNA and AuNPs. This method was selected as it effectively simulates actual wound conditions in an in vitro setting, with phosphate-buffered saline (PBS) applied on top of the gel to mimic topical administration. The use of a beaker setup further enables continuous monitoring of drug release without disrupting the integrity of the gel structure [21].

#### 2.2.11. Sub-acute toxicity study.

***2.2.11.1 Animal care and experimental procedures***. Healthy adult Wistar rats aged 8–9 weeks of both sexes weighing 250–300 g were used for the sub-acute toxicity study of the developed gel. The animals were provided by the Laboratory Animal Resource Unit (LARU), UKM. The rats were housed in individual plastic cages containing wood shavings, that were placed under standard temperature and humidity with a 12-hour light/dark cycle for 7 days for the purpose of acclimatizing the rats to the new environment. Rat pellets were provided as food to the rats and water ad libitum. This experiment received Animal Ethics approval from the UKM Animal Ethics Committee (Approval Number: FF/2021/HALIZA KATAS/22-SEPT./1199-NOV-2021-JUN-2022).

The rats were randomly divided into four groups of four males and four females each and treated as follows:

**Table:** 

Group	Treatment
1	Control (untreated)
2	Positive Control (Intrasite Gel)
3	Blank (Blank Pluronic Gel)
4	Pluronic AuNPs-DsiRNA (Treatment Gel)

***2.2.11.2 Clinical observations and analytical methods***. Rats were visually assessed daily for clinical signs such as skin dehydration, pale eyes, and unkempt fur, alongside behaviour changes like drowsiness and decreased food and water intake. Body weight and food intake were monitored daily for 28 days, with terminal body weight recorded after a 16-hour fasting period to calculate relative organ weights. Blood samples were collected via an intravenous line located in the tail of the rats on days 0, 14, and 28 for haematological and biochemical analysis using a Haematology Analyzer and an ADVIA Chemistry System. Histopathological examination involved fixing skin, liver, and kidney tissues in 10% formalin, staining with H&E, and analysing under light and fluorescence microscopes for structural changes. Major organ weights were measured and expressed as relative percentages of body weight. A skin irritation test was conducted by applying the treatment gel to shaved dorsal areas, with observations recorded at multiple intervals to assess irritation.

#### 2.2.12 *In Vivo* Efficacy of AuNPs-DsiRNA Gel.

***2.2.12.1 Wistar rat diabetic model***. In this study, 32 male Wistar rats aged 8–9 weeks and weighing between 200–300 g were used. The rats were randomly assigned to eight groups, with four rats per group (n = 4/group). The animals were housed under a controlled 12-hour light/dark cycle at room temperature and provided with a standard diet and water ad libitum. Throughout the study, both water intake and animal weight were closely monitored. Before the experiment was started, the rats were acclimated to the laboratory environment for a week. This study was carried out in strict accordance with the recommendations in the Guide for the Care and Use of Laboratory Animals of the National Institutes of Health. The protocol was approved by the Committee on the Ethics of Animal Experiments of the Universiti Kebangsaan Malaysia (Approval Code: FF/2023/HALIZA KATAS/15-FEB./1313-FEB.-2023-DEC.-2023). All surgery was performed under a rat euthanasia of Ketamine, Xylazin and Zoletil, and all efforts were made to minimize suffering.

***2.2.12.2 Diabetes Induction, Wound Creation, and Treatment***. Animals were fasted for 16 hours before receiving a single intraperitoneal injection of freshly prepared STZ (80 mg/kg) dissolved in 0.1M sodium citrate buffer (pH 4.5). After injection, they were given 10% sucrose water for 24 hours. On Day 7, fasting blood glucose levels were measured via tail vein sampling using a VivaCheckTM Eco system. Animals with glucose levels exceeding 8.3 mmol/L (150 mg/dL) were classified as diabetic [22].

Diabetic rats were anesthetized with a KTX mixture (0.2 mL/100 g) intraperitonially, and their dorsal skin was shaved and disinfected. A 10 mm full-thickness wound was created using a biopsy punch, and the initial wound area was photographed for baseline measurement. Treatment was administered as per experimental group protocols, and wounds were covered with Tegaderm Nextcare and Hypafix dressings to maintain a controlled environment.

***2.2.12.3 Fasting Blood Glucose Measurement***. Blood glucose levels were monitored on Days 0, 7, and 11. Blood samples were collected by making a small incision in the tail using a 25-gauge needle and measured using a blood glucose monitoring system.

***2.2.12.4 Wound Closure Measurement.*** Wound areas were recorded on Days 0, 2, 4, 6, 7, 8, 10, and 11 using a camera with a ruler placed beside the wound as a scale. The wound area was analysed and calculated using ImageJ software:


$$Wound\;closure,\;\%=\;\frac{Wound\;area\;Day\;0-\;Wound\;area\;N}{Wound\;area\;Day\;0}\;x\;100$$

Where N is treatment day.

***2.2.12.5 Rats Necropsy***. On days 7 and 11, rats were humanely sacrificed by placing them in a CO₂ chamber. After euthanasia, the wound area was carefully excised. The removed tissue samples were immediately immersed in RNAprotect solution to stabilize and preserve the RNA, ensuring the integrity of the samples for subsequent molecular analysis. The samples were then stored at −80°C in a freezer until further analysis could be performed. This process ensured that the biological material was well preserved for detailed examination of gene expression and other relevant molecular studies.

***2.2.12.6 qPCR for Target Gene Regulation***. Tissue samples (<30 mg) were processed for RNA extraction using the RNeasy Fibrous Tissue Mini Kit, according to the protocol provided by the manufacturer (Qiagen, Germany). The quality and concentration of the extracted RNA were then assessed using a NanoDrop spectrophotometer, ensuring optimal RNA integrity for further analysis.

Subsequently, the isolated RNA was reverse transcribed into complementary DNA (cDNA) using the QuantiTect Reverse Transcription Kit, following the provided protocol (Qiagen, Germany). This step is essential to generate a reliable template for subsequent PCR analysis. For quantitative PCR (qPCR) analysis, the QuantiNova SYBR Green PCR Kit (Qiagen, Germany) was used to measure the expression levels of the targeted genes. This method allows for the precise detection and quantification of gene regulation, enabling a detailed understanding of the molecular mechanisms involved in the wound healing process.

#### 2.2.13 Statistical analysis.

All data are presented as mean ± SEM. Statistical analysis was performed using ANOVA with Dunnett’s post-hoc test, considering results significant at p < 0.05. Data were analysed with IBM SPSS Statistics 29.0.

## 3 Results and discussion

### 3.1 Formation and characterization of gold nanoparticles

#### 3.1.1 AuNPs formation.

AuNPs were formed within a few hours after sonication, as indicated by a change in the color of the solution from yellowish to dark purple, indicating the reduction of gold ions to neutral gold atoms [23,24].In this study, AuNPs were synthesised using a green biosynthesis method involving TMM extract and chitosan as reducing agents and stabilizers, which is more environmentally friendly than chemical methods.

Compounds in the TMM extract drive the nucleation process by reducing Au³⁺ to Au⁰. Chitosan is important as a stabilizing agent, ensuring the appropriate particle size produced. Without chitosan, nanoparticles tend to increase in size and become unstable. When comparing lab-scale and large-scale synthesis, it is evident that large-scale requires shorter sonication times (3–5 hours versus 5–8 hours), as the larger solution volume accelerates nucleation.

**Fig 1:**
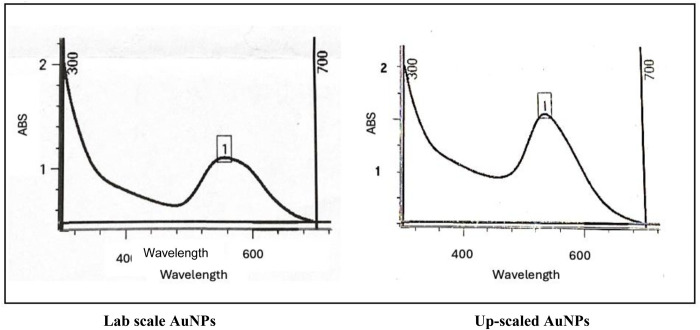
SPR band of biosynthesized AuNPs prepared at lab scale and upscaled.

The presence of AuNPs was confirmed by UV-Vis spectrophotometry (Fig 1), by detecting the surface plasmon resonance (SPR) band at the range from 520 to 580 nm [25]. A higher maximum wavelength was observed for higher extract concentrations, indicating the production of larger particles [26]. This finding aligns with earlier research, which observed a color change in the solution to dark purple after 3–5 hours of incubation. However, extended incubation times may lead to agglomeration [27].

#### 3.1.2 Particle size, PDI and zeta potential.

Based on Table 1, the size of AuNPs decreased as the SPR band intensity increased, influenced by the concentration of the reducing agent and sonication time. The size and morphology of the synthesized AuNPs can be controlled by optimizing the concentration of the reducing agent [28]. In this study, the AuNPs produced at a larger scale were slightly smaller than those produced at a lab scale, likely due to changes in heat transfer, mass, and stabilization conditions. Sonication time also affects the size of the particles produced. Smaller particles are obtained when the sonication time increases from 30 minutes to 1 hour or until a stable reading is reached, indicating the formation of AuNPs. In this study, one formulation showed a decrease in AuNP size from 331 ± 0.9 nm to 251 ± 2.11 nm within 1 hour.

**Table 1:** The particle size, PDI and zeta potential of biosynthesized AuNPs, n = 3.

TMM concentration (0.125 mg/mL)	Particle size (nm) ± SD	PDI ± SD	Zeta potential (mV) ± SD
Lab scale	186.5 ± 0.52	0.3 ± 0.00	+37.6 ± 0.64
Up-scaled	152.9 ± 1.56	0.3 ± 0.00	+37.3 ± 0.26

The zeta potentials of both scales were positively charged (>+30), indicating the stability of the AuNPs suspension due to the protonated amine groups on the chitosan layer, which prevented agglomeration. At acidic and neutral pH, chitosan ensured the stability of the nanoparticles by preventing uncontrolled interactions.

The PDI parameter for both scales was 0.3, reflecting a medium size distribution suitable for drug delivery applications [29]. Statistical analysis showed no significant differences between the scale-up scale and the lab scale for particle size, PDI, and zeta potential.

#### 3.1.3. Morphology.

**Fig 2:**
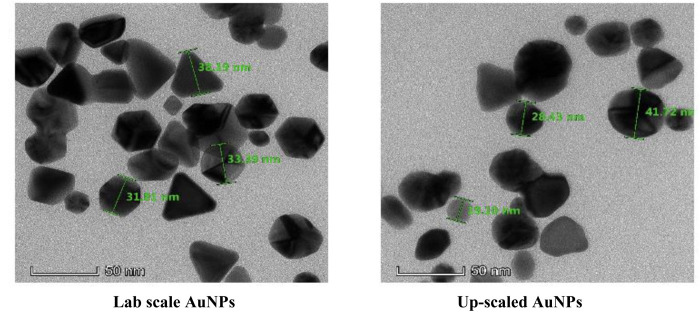
TEM micrograph of lab-scale and upscaled AuNPs at a magnification of 50 nm.

TEM was used to analyse the size and morphology of the synthesized AuNPs. TEM images (Fig 2) showed the core size of AuNPs to be 32.2 ± 7.1 nm (up-scaled) and 37.9 ± 9.3 nm (lab-scale), with most particles being spherical, but triangular, rod-shaped, and hexagonal shapes were also found. This morphological variation depends on the concentration of the TMM extract, which can be optimized for various applications [30,31].

Particle morphology plays an important role in antimicrobial properties and cellular uptake. For example, triangular shapes show the highest uptake by macrophages [32], and the interaction with bacterial cell walls is also influenced by the shape of AuNPs [33]. The lack of biomolecular capping on the particles can lead to irregular shapes and anisotropic nanoparticles [34].

Additionally, the size of AuNPs measured by TEM is smaller than that measured by DLS. This is because TEM captures only the nanoparticle’s metal core without the surrounding hydrated layer, whereas DLS measures the hydrodynamic diameter, which includes both the hydration shell and any surface coatings [35]. The size difference may also be attributed to DLS detecting the presence of chitosan molecules attached to the nanoparticle surface, while TEM visualizes only the electron-dense core [36].

#### 3.1.4. Fourier transform infrared (FTIR) Analysis.

**Fig 3:**
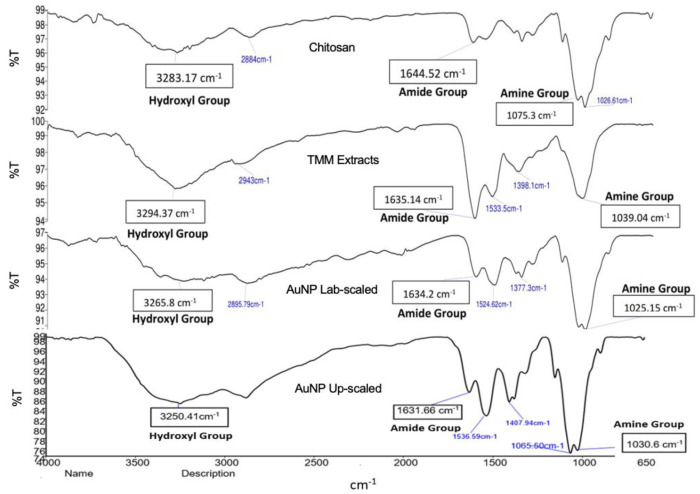
FTIR spectra of AuNPs and their individual constituents (TMM extract and chitosan).

FTIR analysis (Fig 3) was used to identify the biomolecules that stabilized the AuNPs and showed the reduction of gold (III) during synthesis. This helped to identify the chemical bonds between AuNPs and chitosan and the functional groups in the TMM extract that functioned as reducing agents [37].

The FTIR spectra showed -OH peaks at 3283.17 and 3294.37 cm ⁻ ¹ in chitosan and TMM which shifted to 3265.8 cm ⁻ ¹ (up-scaled) and 3250.41 cm ⁻ ¹ (lab scale) after AuNP synthesis, indicating the reduction of Au³⁺ to Au [23,38]. The amide (1644.52 and 1635.14 cm ⁻ ¹) and amine (1075.3 and 1039.04 cm ⁻ ¹) bands in chitosan and TMM also underwent a shift after synthesis, with AuNPs peaks at 1631.66 cm ⁻ ¹ (amide) and 1020.6 cm ⁻ ¹ (amine), indicating biomolecular interactions with AuNPs [39].

This suggests that biomolecules in TMM extracts may be involved in facilitating the formation of AuNPs, mainly through the oxygen function contained in the extract [40]. These results also indicate that TMM extract contains abundant hydroxyl and amide groups, which ensures the success in producing AuNPs [41]. The presence of proteins on the surface of AuNPs synthesized at lab and scale-up scales can be represented by bonds related to amide groups (N–H). Functional groups found in heterocyclic compounds such as alkaloids or flavones, and the major amide bonds found in proteins are capping ligands for nanoparticles [42]. FTIR analysis confirmed that AuNPs can be synthesized and effectively capped using TMM extract and chitosan.

### 3.2. Statistical process optimization of AuNPs production using response surface methodology (RSM)

CCD was used as an RSM optimization tool to evaluate and optimize the effects of the HauCl4 and TMM extract concentration variables and the wavelength absorption of AuNPs as a reaction product. RSM is a combination of statistical and mathematical tools to design experiments and optimize process variables and effects minimizing the number of trials. In this study, RSM assisted in achieving high AuNPs yields by identifying the influence of process parameters [43]. The methodology involves a two-step process: modelling and optimization. In the modelling step, first or second order polynomial equations are fitted to the experimental data, followed by analysis of variance (ANOVA) to assess the validity of the model. Once validated, the model can be visualized using a three-dimensional response surface plot, which helps identify the optimal operating conditions for the process [44].

#### 3.2.1. RSM modelling and analysis.

**Table 2** shows the CCD design for the two parameters along with the ‘yield’ (Absorption) of AuNPs as the response. The coded levels of the independent variables are provided in the S1 Table in S1 File. The highest response, 0.048 was found in (run 10), while the lowest response was found in run 8 which was 0.007.

**Table 2:** CCD Design for two variables with AuNP yield as the response.

Run	A: HauCl_4_ Conc. (mL)	B: TMM conc. (mL)	Response Yield: Absorbance (nm)
1	0.6	3.2	0.023
2	0.8	4.35	0.021
3	0.8	4.2	0.019
4	0.7	5.97	0.035
5	0.6	5.5	0.035
6	0.7	4.35	0.016
7	0.7	4.2	0.017
**8**	**0.7**	**2.723**	**0.007**
9	0.8	5.5	0.016
**10**	**0.56**	**4.35**	**0.048**

In the optimization experiment, 10 runs were conducted based on the Central Composite Design (CCD). ANOVA predicted a second-order polynomial function to describe the relationship between variables. The regression equation, expressed in coded factors, is as follows:


$$(\text{AuNPs yield})=\text{ 0}.\text{0162}-\text{0}.\text{006}{\text{6}}^{*}\text{A }+\text{ 0}.\text{001}{\text{5}}^{*}\text{B }-\text{ 0}.\text{003}{\text{6}}^{*}\text{AB }+\text{ 0}.\text{011}{\text{1}}^{*}\text{A2 }-\text{ 0}.\text{004}{\text{1}}^{*}\text{B2}$$ Equation 1

Statistical analysis of Equation 1 showed that positive values have a synergistic effect on the reaction (AuNPs yield) and negative values represent an antagonistic effect on the reaction, where A and B are the concentration of HAuCl4 and the concentration of TMM extract, respectively. Based on the coefficient values of this equation, it is clear that the concentration of TMM extract has a higher positive effect than the concentration of gold chloride (HAuCl4) in achieving high AuNPs yield.

#### 3.2.2. Interpretation of RSM three-dimensional surface plots.

**Fig 4:**
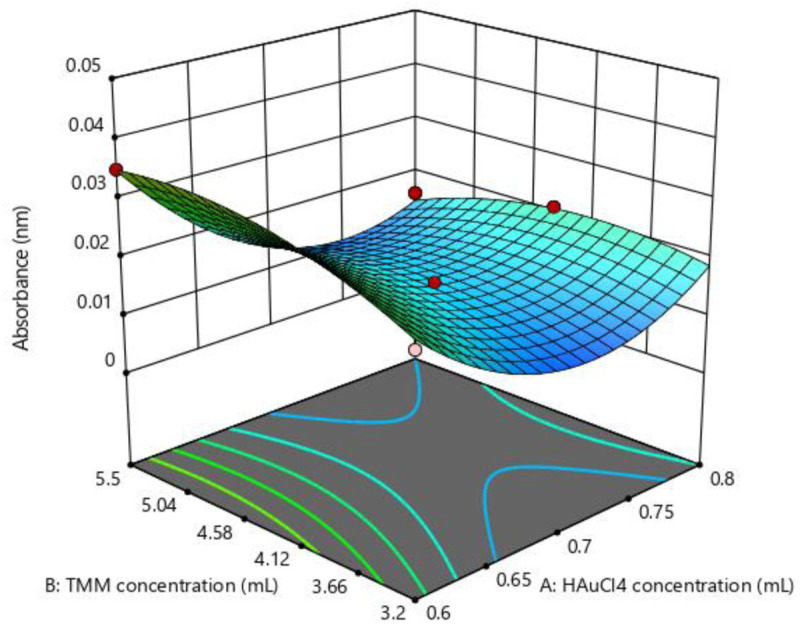
3D plot of AuNP results using the interaction of HAuCl₄ concentration and TMM.

3D surface response plots were generated to explore the interaction between HAuCl₄ and TMM concentrations and to identify optimal conditions for maximizing AuNP yield (Fig 4). The plots confirmed that the yield depends more on TMM concentration.

**Fig 5:**
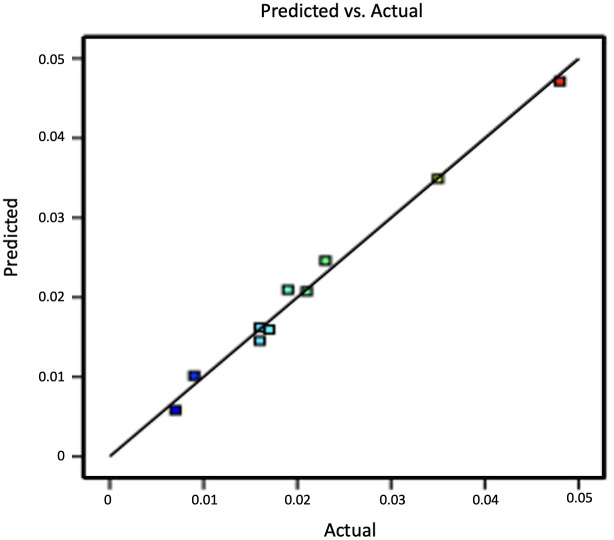
Predicted vs. Actual plot.

The “Predicted vs. Actual” plot (Fig 5) demonstrated the model’s accuracy, with data points aligning closely to a 45-degree line. This alignment, along with the absence of response transformation or normality issues, validates the model’s robustness and reliability in predicting AuNP yield.

#### 3.2.3 Experimental Model Validation.

**Fig 6:**
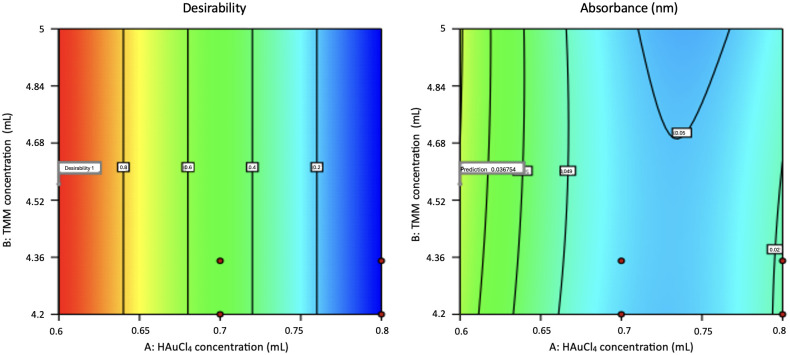
Contour plot for optimal AuNP synthesis parameters.

Fig 6 shows the Contour plot of the parameters used to optimize the synthesis of AuNPs in an attempt to achieve higher yields. The results of the model proposed by RSM were validated by conducting an experiment using parameters according to the optimal production conditions of AuNPs synthesis. The optimization of the variable parameters was carried out in a numerical optimization method.

A total of 20 solutions were found from the two-stage factorial design optimization. The optimal values for each variable are shown as follows: A = 0.6 and B = 4.565 and the model predicted that the synthesis yield of AuNPs was 0.035 and the experiment was 0.043, which was higher than the predicted value. This indicates the potential to achieve higher yields, giving a positive sign in the synthesis process.

### 3.3. DsiRNA entrapment and binding efficiency

**Fig 7:**
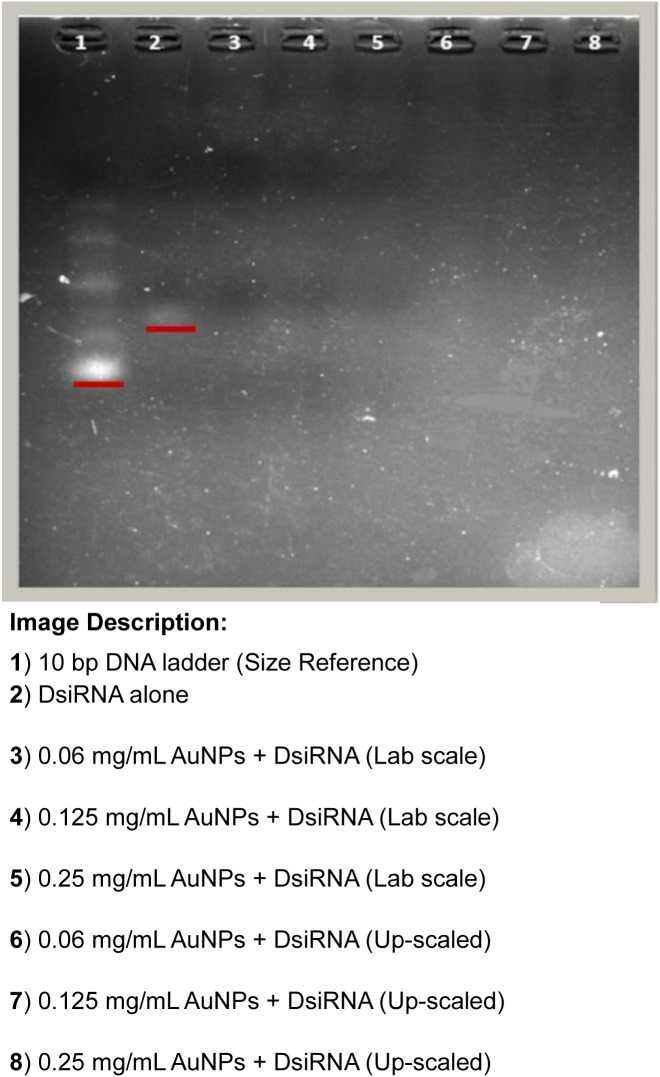
Gel electrophoresis of AuNPs-DsiRNA at different concentrations. AuNPs were produced using lab-scale and scaled-up.

Different concentrations of AuNPs were used for DsiRNA adsorption (0.06, 0.125 and 0.25 mg/mL) onto the AuNPs surface. All concentrations of AuNPs tested were found to bind DsiRNA strongly with an entrapment efficiency (EE) value of more than 70% (Table 3). This was supported by the gel electrophoresis results used to evaluate the binding efficacy (BE), where no DsiRNA tail bands were observed, indicating the absence of DsiRNA release from AuNPs nanoparticles (Fig 7). The strong binding of DsiRNA to AuNPs allowed the active dose to be effectively delivered to the target site with high EE [45]. Furthermore, similar EE values were also obtained for both lab and scale-up scales.

**Table 3:** Effect of AuNPs concentration on the percentage encapsulation efficiency (EE) for DsiRNA, n = 3.

AuNP Types	AuNPs conc., mg/mL	EE (%)
Up-scaled	0.06	71.1 ± 0.44
	0.125	74.1 ± 0.32
	0.25	72.0 ± 0.28
Lab scale	0.06	76.3 ± 0.1
	0.125	70.4 ± 0.1
	0.25	74.0 ± 0.36

### 3.4. Antibacterial assay

**Fig 8:**
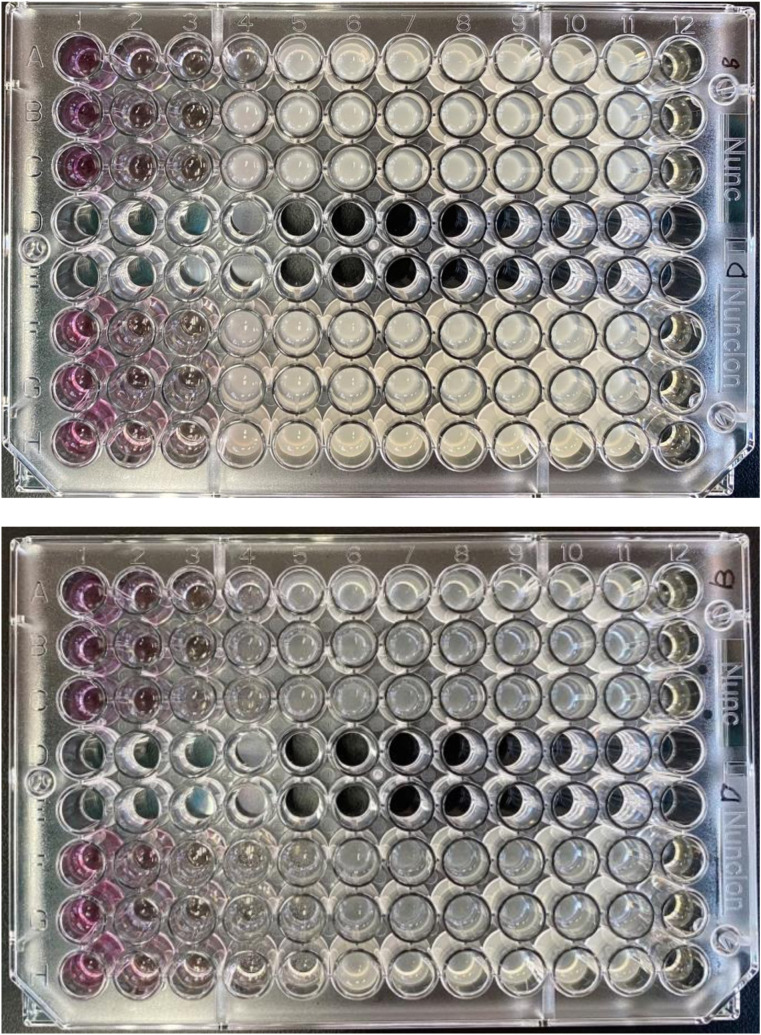
MIC for AuNPs against *S. aureus* (top) and *P. aeruginosa* (bottom) as determined in the micro-broth dilution test, n = 4.

The antibacterial activity of AuNPs was tested against *P. aeruginosa* and *S. aureus* with an MIC value of 250 μg/mL, showing efficacy against both gram-positive and gram-negative bacteria (Fig 8). In this assay, the growth control (GC) consisted of bacteria in broth to confirm viability, while the sterility control (SC) contained only broth without bacteria to confirm aseptic conditions. The thinner cell walls of gram-negative bacteria allow AuNPs to penetrate the cell membrane more easily than those of gram-positive bacteria, which have thicker cell walls. Optimal particle size facilitates cell wall penetration, while large particles due to aggregation may reduce antibacterial efficacy.

Chitosan coating of AuNPs enhances antibacterial activity through the protonated positive charge of NH₃ ⁺ , which interacts strongly with the negatively charged bacterial cell membrane [46,47]. The smaller particle size also contributes to efficacy, providing a larger surface area for interaction with bacteria [48]. This interaction results in membrane damage, leakage of cell contents, and DNA damage, ultimately leading to bacterial cell death [49,50]. Previously, varying the sizes of AuNPs from 80.3 ± 23.4 nm to 125.41 ± 41.5 nm by reducing the TMM extract concentration from 0.05 to 0.0125 g/mL did not significantly impact the antibacterial activity against *S. aureus*, *P. aeruginosa*, and *E. coli* (Katas et al., 2019).

### 3.5 Physical characterization of thermoresponsive gels

#### 3.5.1 T_gel_.

T_gel_ is the sol-gel transition temperature, which is the temperature at which a gel changes from a liquid phase to a gel. This property is important for biomedical applications, such as in situ gels that are easily applied topically in a liquid state at room temperature and form a protective layer at body temperature [51].

The ideal T_gel_ for topical application is usually between 25°C and 37°C, ensuring that the gel remains liquid during application and transforms into a gel at the target site [52]. In this study, the T_gel_ for the scale-up and lab formulations were around 31°C ± 1 and 28°C ± 3, respectively, making them suitable for biomedical applications [17].

This thermoresponsive gel provides a protective barrier, bio-adhesiveness, and optimal moisture at the wound site, accelerating healing and reducing the risk of infection [53]. The polymer structure ensures that it remains at the application site while promoting tissue regeneration.

#### 3.5.2. Rheology.

**Fig 9:**
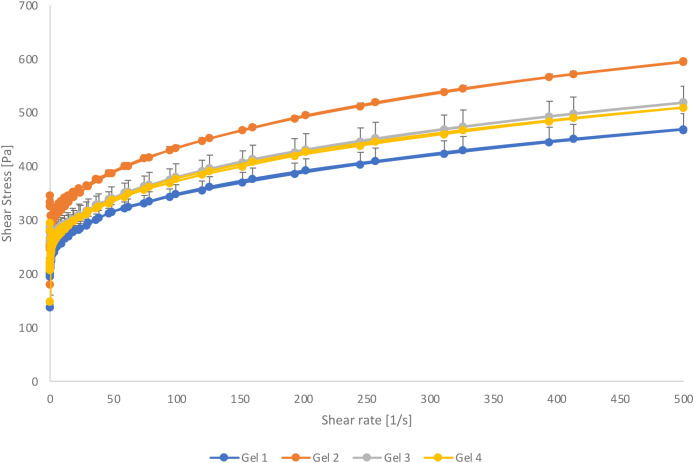
Rheological profile of PF127 gel formulations at different scales. (1) Blank Pluronic gel (Upscaled). (2) Blank Pluronic gel (Lab scale). (3) Pluronic gel with AuNPs-DsiRNA (Upscaled). (4) Pluronic gel with AuNPs-DsiRNA (Lab scale). n = 3.

Rheometer analysis was conducted to evaluate the flow and deformation behaviour of the gel formulations [54] with a focus on monitoring the flow curves and identifying non-Newtonian effects at controlled temperatures. As illustrated in Fig 9, all formulations exhibited shear thinning behavior (p > 0.05), indicating pseudoplastic or non-Newtonian properties, which can be attributed to their thermoresponsive properties [17,55]. The observed decrease in viscosity with increasing shear rate underscores the ability of the gel to adapt to varying stress conditions, which is important to ensure that it remains intact at the wound site during application.

The gels produced through scale-up showed lower viscosity compared to the lab-scale gels, however, they maintained sufficient viscosity for wound healing applications. The decrease in viscosity may be attributed to the higher content of ingredients such as TMM extract and chitosan, which can disrupt the micelle structure of Pluronic F127. This behavior may occur where micelles form a cubic lattice microstructure that collapses and flows under applied stress, consistent with microstructural observations [56]. The shear-steady behavior of these formulations is influenced by both temperature and polymer content [57]. Solidification of all gel formulations with increasing temperature is desirable as it ensures that the gel remains intact at the wound site once applied.

#### 3.5.3 TPA.

Texture profile analysis (TPA) was conducted to evaluate the hardness, cohesiveness, and stickiness of Pluronic gels containing AuNPs-DsiRNA, with the aim of optimizing the formulation for topical application in wound treatment. The desired hardness and cohesiveness are low, allowing for easy removal from the container and smooth application. This low hardness also ensures that the gel remains intact on the skin and forms a stable protective layer over the wound. Increasing the polymer concentration, however, tends to increase the hardness and cohesiveness values of the gel, directly correlating with the increase in viscosity [58]. Adhesiveness, on the other hand, is an important property related to the work required to remove the gel from the surface to which it is attached. High adhesion is preferred, as it can prolong the gel retention time on the skin, allowing better absorption of the active ingredient and also maintaining a moist environment to promote wound healing [59].

Overall, the data in Table 4 show that the scaled-up gels maintain important properties despite slight physical differences, such as cohesiveness and hardness, without significant differences (p > 0.05), indicating the gel’s potential for commercialization and further application in the field of wound healing.

**Table 4:** TPA (Hardness, Cohesion and Adhesion) of PF127 gels blank and containing AuNPs-DsiRNAs on up-scaled, and lab scale (n = 3).

Group	Hardness (N)	Adhesiveness (mJ)	Cohesiveness (mJ)
Blank Pluronic (Up-scaled)	5.77 ± 0.18	18.2 ± 0.83	1.33 ± 0.04
Blank Pluronic (Lab scale)	7.60 ± 0.07	22.47 ± 0.27^*^	1.29 ± 0.02
AuNPs-DsiRNA (Up-scaled)	6.48 ± 0.69	19.4 ± 0.44	1.24 ± 1.08
AuNPs-DsiRNA (Lab scale)	5.15 ± 0.35	17.28 ± 0.98	1.28 ± 0.04

Footnote: * p < 0.05 statistically different from the Blank Pluronic (Up-scaled) group

Optimal formulations require a balance between cohesiveness and cohesion. Increased adhesion supports wound coverage but may compromise gel integrity [60]. These textural properties provide guidance for producing effective and consistent formulations in real wound healing applications [61].

### 3.6 Drug release profile

**Fig 10:**
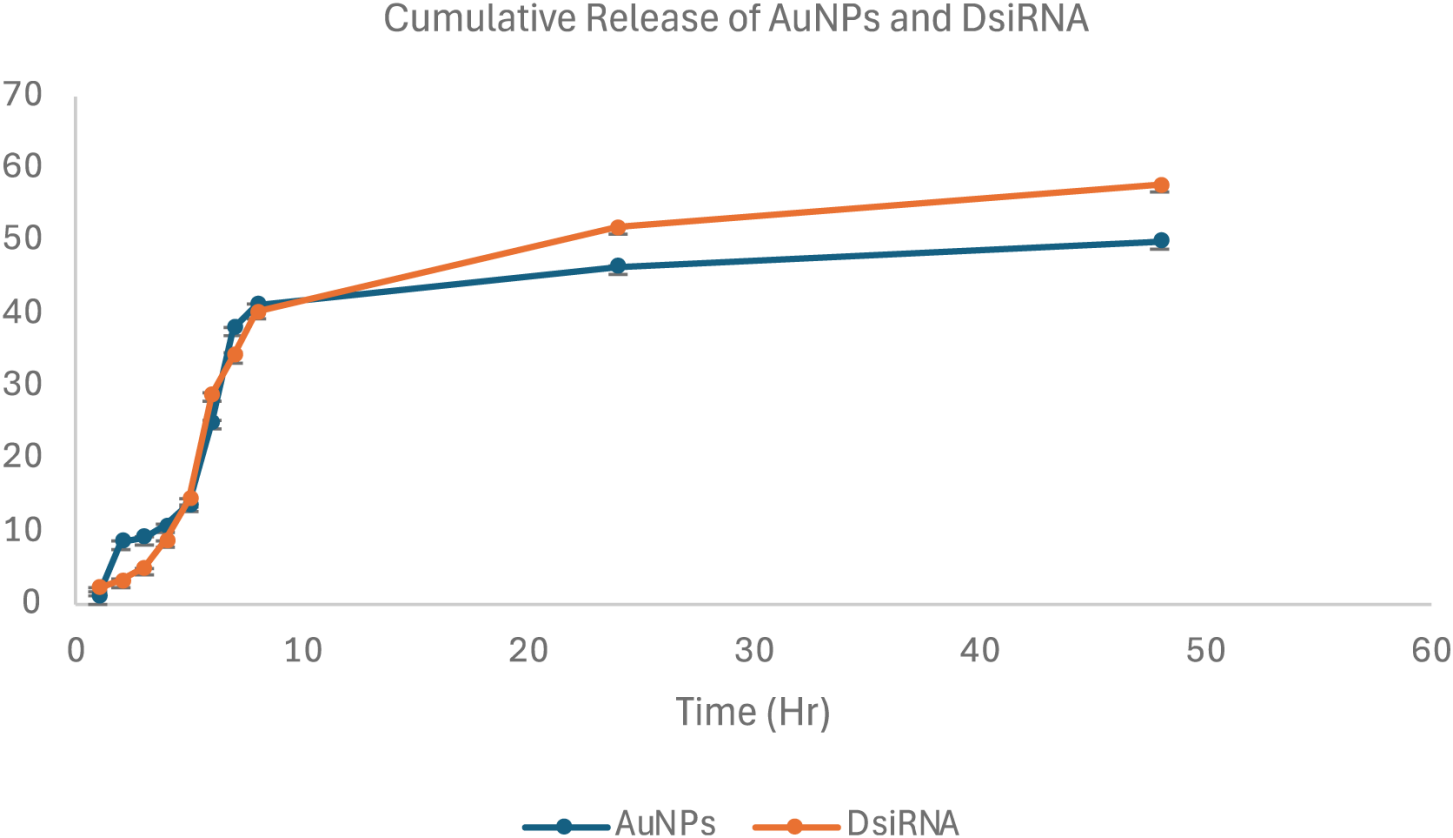
Cumulative (%) release of AuNPs and DsiRNA from the thermoresponsive gel at pH 7.4 and 37 °C for 48 hours, n = 3.

Drug release profiling was conducted to evaluate the release of AuNPs-DsiRNA from the optimized and scaled-up PF127 gels, with release through PBS over 48 hours (Fig 10). Initially, a burst release occurred before transitioning to a sustained phase, which ensured that therapeutic levels of the active agent were maintained for a longer period, reducing the need for repeated applications [62]. Similar findings have been reported in previous studies [19], indicating that gel properties influence the release rate.

Penetration of nanoparticles into the skin is a complex process and is influenced by various factors, including skin type, barrier integrity, and physicochemical properties of the nanoparticles such as size, shape, and surface charge [63]. These properties, in particular the positive surface charge of chitosan-AuNPs, play an important role in increasing skin permeability. The negative charge of the skin under physiological conditions facilitates the interaction between the skin and the positively charged nanoparticles through electrostatic attraction [64]. This combination provides an immediate therapeutic effect and sustained release for long-term efficacy, increasing patient convenience and reducing treatment costs.

### 3.7. Sub-acute skin toxicity of PF127 gel

#### 3.7.1. Safety and efficacy evaluation of AuNPs-DsiRNA Gel.

The increasing demand for AuNPs for therapeutic applications necessitates a comprehensive evaluation of their safety and efficacy. Clinical signs, histological analysis, and blood parameters were carefully studied to confirm these findings. The earliest indicator of potential adverse effects of a chemical or drug often involves changes in body weight [65].

In this study, the topical application of AuNPs-DsiRNA gel on test rats over 28 days showed no adverse effects, as the general behavior of the rats remained normal. There were no significant differences in body weight, food intake, or water consumption compared to the control group. Treated rats exhibited gradual weight gain over the 4-week treatment period, with no signs of stress observed. Additionally, relative organ weights in the treated group were not significantly different (p > 0.05) from the control group. The absence of edema, atrophy, or hypertrophy in the organs further supports the non-toxic nature of the gel [66–68]. These findings suggest that the gel is safe for use without inducing systemic toxicity.

#### 3.7.2 Histological assessment of liver injury.

**Fig 11:**
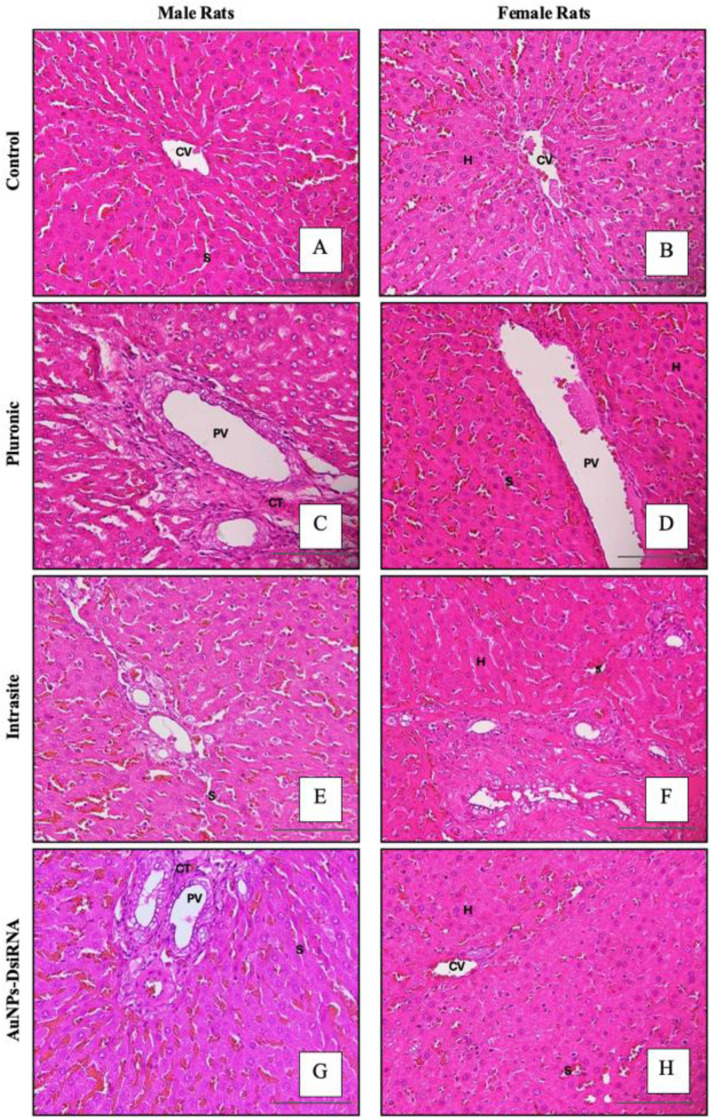
Histopathology of liver in male and female rats. Photomicrographs show histological sections of the liver with hepatocytes (H), sinusoids (S), central vein (CV), portal vein (PV), and connective tissue (CT), (H&E X100).

Liver injury was carefully assessed by histological examination (Fig 11). In both male and female control groups of rats, liver cells exhibited a normal liver structure characterized by polyhedral hepatocytes, containing spherical nuclei, and occasionally binucleate nuclei. These hepatocytes, which are important in toxicological studies due to their role in metabolism and detoxification, were arranged in plates bordered by blood-filled sinusoids [69–71].

In the treated groups, moderate presence of erythrocytes within the sinusoids was noted in the AuNPs-DsiRNA group (male rats). However, this remained within normal histological limits and was not associated with tissue damage or [70,72]. Therefore, the liver histology of the AuNPs-DsiRNA gel treated group showed no obvious differences compared to the untreated control, indicating that the AuNPs-DsiRNA gel did not cause any distinct pathological changes in the liver tissues.

Although the histological results provided strong indications that the gel was not toxic to liver tissues, these findings alone were not sufficient to completely rule out potential toxicity. To support the histological observations, blood parameters were also monitored, providing a more comprehensive assessment of the safety profile of the gel.

#### 3.7.3. Blood analysis to assess toxic effects.

Assessing the effects of exogenous components on blood is important, as blood analysis provides a critical insight into the physiological, nutritional and pathological status of the organism [73,74]. Haematological parameters, in particular, are important indicators to determine the severity of any harmful effects on blood components.

In this study (Table 5), a slight decrease in WBC count was observed on day 28 in male rats treated with AuNPs-DsiRNA gel. However, this decrease was not statistically significant when compared to day 0 and the untreated control group, indicating that AuNPs-DsiRNA gel did not cause toxicity that could lead to leukopenia [75]. This finding is important because leukopenia can negatively affect other blood parameters, such as RBC production.

**Table 5:** Haematological parameters in male and female rat during treatment (0, 14 and 28 days).

	Day	Male				Female			
		Control	Pluronic	Intrasite	AuNPs-DsiRNA	Control	Pluronic	Intrasite	AuNPs-DsiRNA
RBC (x10^12^/L)	0	7.84 ± 0.17	7.36 ± 0.52	7.69 ± 0.62	6.69 ± 0.88	7.68 ± 0.2	6.27 ± 0.16	7.56 ± 0.23	7.09 ± 0.68
	14	7.0 ± 0.76	7.2 ± 0.81	8.09 ± 0.29	8.55 ± 0.32	7.74 ± 0.26	6.99 ± 1.14	8.24 ± 0.05	7.47 ± 0.53
	28	10.14 ± 0.12	9.77 ± 0.09	9.09 ± 0.52	9.67 ± 0.19	7.57 ± 0.46	6.57 ± 0.63	7.25 ± 11.25	7.82 ± 0.39
Hb (g/L)	0	148.67 ± 0.88	135.67 ± 0.56	141 ± 7.0	123.33 ± 17.7	139 ± 1.0	132 ± 9.0	144 ± 3.0	144 ± 5.0
	14	121.5 ± 11.8	125 ± 13.6	147.75 ± 5.45	150 ± 6.36	141.5 ± 7.5	128 ± 19.0	157.5 ± 3.5	134.5 ± 6.5
	28	176.33 ± 3.71	176.33 ± 2.67	167.0 ± 12.66	175.0 ± 3.21	144.0 ± 9.63	122.0 ± 11.47	136.5 ± 1.55	146.0 ± 6.76
PCV (L/L)	0	0.35 ± 0.02	0.36 ± 0.15	0.37 ± 0.15	0.34 ± 0.02	0.36 ± 0.05	0.33 ± 0.05	0.35 ± 0.1	0.35 ± 0.25
	14	0.35 ± 0.04	0.34 ± 0.05	0.39 ± 0.01	0.41 ± 0.02	0.36 ± 0.01	0.34 ± 0.03	0.42 ± 0.0	0.37 ± 0.03
	28	0.47 ± 0.02	0.48 ± 0.02	0.45 ± 0.04	0.48 ± 0.01	0.38 ± 0.03	0.34 ± 0.035	0.38 ± 0.01	0.40 ± 0.03
MCV (fL)	0	44.33 ± 1.45	49 ± 1.52	48 ± 2	52.33 ± 3.67	45 ± 1	50.5 ± 1.5	46.5 ± 0.5	45.5 ± 2.5
	14	49.75 ± 1.11	46.5 ± 3.43	49.0 ± 0.41	47 ± 1.1	46.5 ± 0.5	49.5 ± 3.5	51 ± 0	49.5 ± 0.5
	28	46.33 ± 2.02	48.67 ± 0.67	49.33 ± 1.45	49.33 ± 0.33	50.0 ± 1.29	51.75 ± 0.63	52.75 ± 0.63	51.25 ± 1.31
MCHC (g/L)	0	376.7 ± 44.54	376.7 ± 3.17	369.3 ± 19.53	355.7 ± 29.78	394.5 ± 5.5	391 ± 7	413.5 ± 1.5	395 ± 8.0
	14	351.5 ± 5.17	379.8 ± 30.44	374.3 ± 10.35	371.5 ± 7.75	376.0 ± 27.0	441.5 ± 44.5	375 ± 8.0	364.5 ± 11.5
	28	376.0 ± 10.6	370.0 ± 2.51	371.3 ± 2.19	364.7 ± 2.33	380.5 ± 7.38	360.0 ± 4.14	357.5 ± 6.51	367.5 ± 14.29
WBC (x10^9^/L)	0	17.63 ± 0.95	13.03 ± 1.49	10.93 ± 1.55	13.07 ± 2.03	12.3 ± 0	7.9 ± 0.85	9.55 ± 2.1	8.40 ± 1.56
	14	11.4 ± 1.9	9.4 ± 2.07	12.67 ± 1.03	12.75 ± 0.59	9.7 ± 0.4	8.0 ± 2.1	10.7 ± 0.8	8.25 ± 2.15
	28	9.9 ± 0.4	10.17 ± 1.14	8.13 ± 2.21	9.7 ± 1.67	8.35 ± 1.19	7.82 ± 2.44	8.15 ± 1.13	7.55 ± 1.49

Additionally, the main haematological variables, RBC count, Hb levels, PCV, MCV, and MCHC remained the same as day 0 and were not significantly different from control rats (p > 0.05). Throughout the experimental period, there was no evidence of RBC destruction or changes in erythropoiesis (RBC formation), indicating that this gel had no adverse effects on the overall blood health of the treated animals [66,73]. Although an increase in Hb was observed in the male group, a similar trend was noted in the untreated control group. This could be attributed to factors unrelated to the formulation, such as dehydration, indicating the non-toxicity of AuNPs-DsiRNA gel.

#### 3.7.4. Assessment of hepatic enzyme markers.

The liver plays a key role in drug metabolism and is often a target for toxic [76]. In this study (Table 6), the potential for liver damage was assessed through the main hepatic enzyme markers: AST, ALT, and ALP. These enzymes are released into the bloodstream upon hepatocellular injury [77,78].

**Table 6:** Blood biochemical parameters for liver function of treated male and female rats (0, 14 and 28 days), n = 4.

	Day	Male				Female			
		Control	Pluronic	Intrasite	AuNPs-DsiRNA	Control	Pluronic	Intrasite	AuNPs-DsiRNA
ALP (U/L)	0	160 ± 48.6	289.5 ± 22.9^*^	240 ± 32.4	213 ± 17.7	226 ± 75.9	262.7 ± 4.5	153.5 ± 29.1	183.3 ± 23.7
	14	239.8 ± 46.7	277.5 ± 40	317.25 ± 61.2	268.5 ± 25.4	187.7 ± 66.8	273 ± 44.5	219 ± 12.2	150.7 ± 31.5
	28	119 ± 22.7	154.7 ± 10.1	128.3 ± 34.5	131.3 ± 33.6	106.8 ± 15.6	116.3 ± 26.0	93.5 ± 20.5	73.3 ± 15.9
AST (U/L)	0	331.5 ± 48.4	308 ± 23.5	269.8 ± 53.9	198 ± 12.1^*^	235 ± 22.8	380 ± 49.9	233 ± 59.4	193.5 ± 8.4
	14	251.5 ± 31.5	255.8 ± 27.9	219 ± 50.9	189.5 ± 46.7	210.7 ± 32.9	284.3 ± 1.8	145.7 ± 33.9	159.3 ± 39.1
	28	278.3 ± 51.7	253.3 ± 38.4	184 ± 29.9	215.3 ± 21.7	171.8 ± 30.5	250.8 ± 28.7	231.3 ± 12.4	225.8 ± 32.8
ALT (U/L)	0	90 ± 5.4	83 ± 13.9	87.3 ± 28.9	66.5 ± 4.5	55.5 ± 12.7	92.5 ± 27.1	51.5 ± 12.3	61.8 ± 6.1
	14	80.8 ± 5.9	90.3 ± 10.8	83.3 ± 3.3	79 ± 5.8	47.3 ± 7.8	71.7 ± 0.9^*^	64 ± 0.6	42.7 ± 5.4
	28	58.5 ± 5.5	63.3 ± 8.8	47 ± 1.2	50.8 ± 2.1	45 ± 5.7	70.25 ± 7.6	64 ± 6.8	49 ± 8.1

Footnote:

* p < 0.05 statistically different from the Control group

The results showed that ALP and ALT levels decreased on day 28 compared to day 0, while AST levels increased slightly but not statistically significantly (p > 0.05). No significant differences were also observed between the treated and untreated control groups, indicating the absence of hepatic injury [71].

This finding is supported by the fact that the size of AuNPs used (>100 nm) reduced the risk of toxicity, as larger particles were less likely to penetrate liver cells. In contrast, smaller nanoparticles (<60 nm) are more likely to cause liver damage, as demonstrated by increased ALT and AST [79].

The normal reference ranges for ALP, AST, and ALT in rats were obtained from [80] and were used as a benchmark for evaluating potential hepatotoxicity. Although there were changes in the levels of ALP, AST, and ALT, with some values falling outside the normal range, these changes were not significantly different from Day 0 and the untreated control groups. This indicates that the AuNPs-DsiRNA gel did not cause liver toxicity. It is important to note that natural biological variability among individual rats can lead to some having higher baseline liver enzyme levels. Therefore, comparing enzyme values with the untreated control group provides a more accurate and meaningful assessment of hepatic safety. Additionally, since clinical biochemical analyses can vary due to methodological differences, using the untreated group as a direct experimental control offers a more consistent and reliable benchmark than depending solely on standard reference values [81].

#### 3.7.5. Assessment of renal function and potential toxicity.

The potential toxic effects of AuNPs-DsiRNA gel on renal function can be assessed by analysing the levels of urea and creatinine in blood serum [77]. Based on Table 7, a slight increase in creatinine was observed on day 14, but by day 28, the levels had returned to normal when compared to day 0. The results for both urea and creatinine at Days 14 and 28 did not show significant differences when compared to the control group and Day 0, indicating no renal injury or abnormalities in renal function.

**Table 7:** Blood biochemical parameters for kidney function of male and female rats during treatment (0, 14 and 28 days).

	Day	Male				Female			
		Control	Pluronic	Intrasite	AuNPs-DsiRNA	Control	Pluronic	Intrasite	AuNPs-DsiRNA
Creatinine (μmol/L)	0	37.5 ± 0.9	36.8 ± 1.5	29 ± 4.4^*^	28.8 ± 1.5	41.8 ± 4.3	33.5 ± 2.4	26.8 ± 5.5	29.5 ± 0.9
	14	48 ± 11.7	45.8 ± 1.4	45.5 ± 17	40.5 ± 3.9	34.3 ± 8.9	33.3 ± 28.2	41.3 ± 16.9	40.6 ± 1.9
	28	34.3 ± 6.9	34.5 ± 3.6	24 ± 7.9	32.8 ± 6.6	39 ± 1.8	28 ± 5.9	34.3 ± 5.9	30.5 ± 3.6
Urea (μmol/L)	0	5.3 ± 0.2	6.6 ± 0.8	6.3 ± 0.6	6.4 ± 0.4	4.4 ± 1.2	5.4 ± 0.3	3.9 ± 0.8	4.7 ± 0.4
	14	5.7 ± 0.4	5.3 ± 0.5	5.2 ± 0.2	5.7 ± 0.2	4.4 ± 1.2	6.1 ± 0.1	5.9 ± 3.8	5.7 ± 2.0
	28	5.6 ± 0.5	6.3 ± 0.2	4.9 ± 0.6	5.6 ± 0.9	7.2 ± 0.5	9.2 ± 0.7	7.4 ± 1.3	7.2 ± 1.5

Footnote:

* p < 0.05 statistically different from the Control group)

Nephrotoxicity in rats can be evidenced by elevated blood urea nitrogen (BUN) and creatinine levels [82]. An increase in BUN and creatinine levels suggests a decline in kidney function and may be associated with renal damage. These elevated levels can also lead to renal tissue injury, which may indicate toxicity or kidney failure [83,84]. Comparison was made against both the untreated control group and standard reference ranges as benchmarks to evaluate any treatment-induced changes. All observed values remained within the normal reference range, indicating no toxic effects on renal function [80]. Therefore, these findings can be further validated through histopathological examination of kidney tissue.

#### 3.7.6 Kidney Histopathological Analysis and Observation.

**Fig 12:**
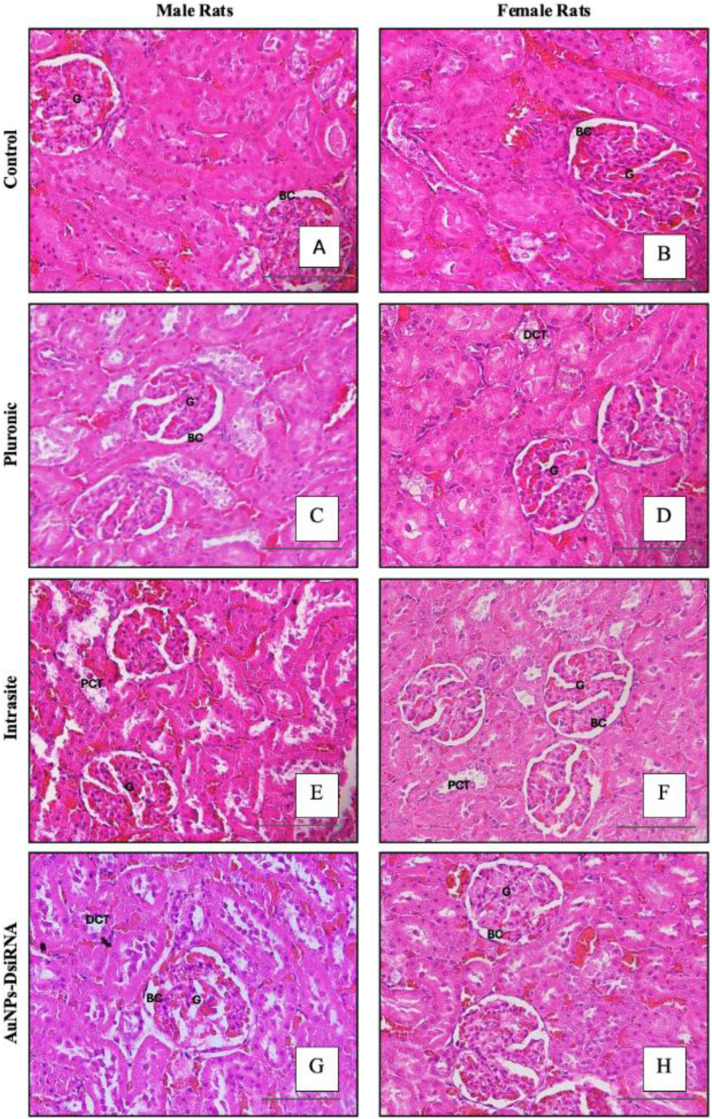
Histopathology of kidney in male and female rats. Photomicrographs show histological sections of the kidney sections that include Bowman’s capsule (BC), proximal convoluted tubule (PCT), distal convoluted tubule (DCT), and glomerulus (G), (H&E X100).

Further confirmation by histopathological examination of kidney tissues using H&E staining confirmed the absence of renal toxicity due to the use of this gel (Fig 12). The kidney morphology of all groups showed normal tissue structure, with intact glomeruli and renal tubules, similar to the untreated control group. No evidence of renal toxicity, such as tubular vacuolization, mononuclear cell infiltration, focal necrosis, or hemorrhage was observed [77,82].

Histopathological examination revealed that the AuNPs-DsiRNA-treated group did not show significant differences when compared to the control and other experimental groups. Mild erythrocyte congestion in the renal tubules was detected but this condition might be attributed to external factors rather than the treatment itself. These factors included the repeated use of KTX as a sedative agent during blood collection procedures, which could cause stress in the rats. Such stress may have influenced blood and tissue histological parameters, leading to slight variations unrelated to treatment [65].

Although liver dysfunction can lead to the accumulation of toxins that the kidneys must filter, potentially causing kidney stress or damage, and acute kidney injury can cause liver stress and elevated liver enzymes due to systemic inflammation and the release of proinflammatory cytokines [85] this was not observed in the study. The changes in liver enzymes, creatinine, and urea were not statistically significant compared to Day 0 and the untreated control group. Furthermore, histopathological anomalies were not detected, confirming no liver and renal injuries.

#### 3.7.7 Skin histological analysis.

**Fig 13:**
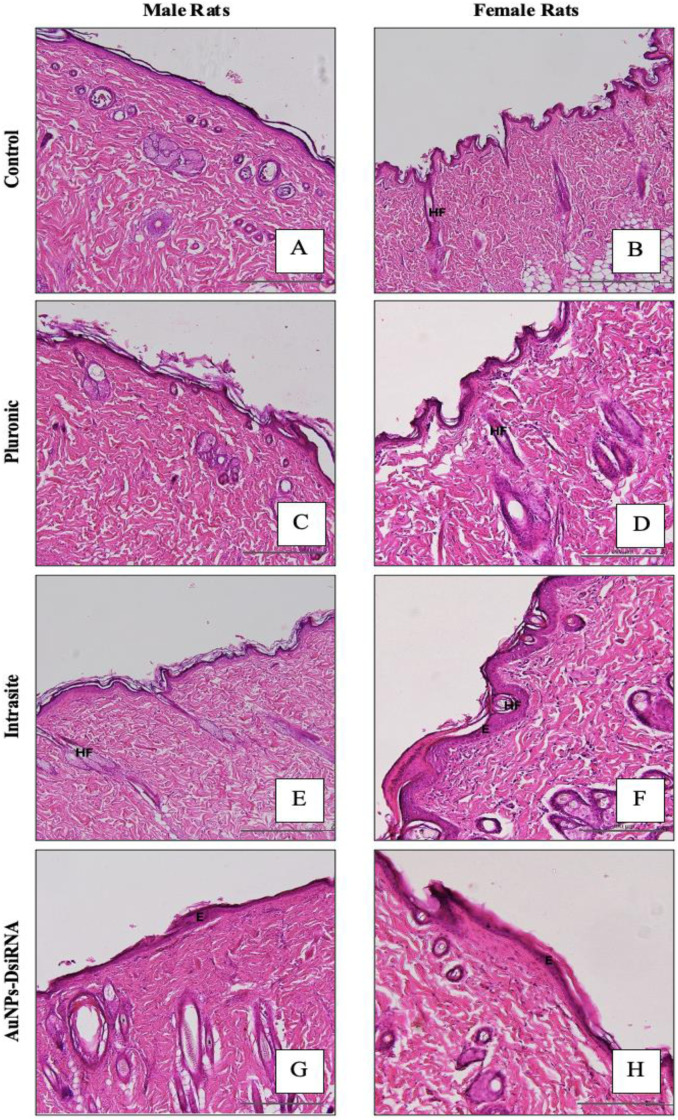
Histopathology of skin in male and female rats. Photomicrographs show histological sections of the skin sections that display the epidermis (E) and hair follicle (HF), (H&E X100).

In skin histological analysis (Fig 13), the AuNPs-DsiRNA treatment groups of both sexes showed comparable results to the other groups. Epidermal integrity remained intact, and cross-sections of hair follicles were visible in the dermis. No significant histological abnormalities were observed, and there was no thickening of the epidermis, which would suggest ongoing inflammation. In addition, no lymphocytic infiltration or inflammatory cells were detected, indicating the absence of any active immune response or inflammation [86,87].

#### 3.7.8. Gender influence on AuNPs-DsiRNA Toxicity.

The use of male and female rats in toxicity studies is important because gender differences are known to affect sensitivity to certain substances. Female rats, in particular, may be more susceptible due to differences in hormones and how the body regulates and metabolizes these substances [67,68].

In this study, blood test results for male and female rats showed comparable results. For example, ALT readings for both sexes followed the same trend from days 0, 14, and 28, indicating no significant toxicity to AuNPs-DsiRNA based on gender. This suggests that AuNPs do not exert different toxic effects based on the sex of the rats. Furthermore, histological images of the liver and kidneys of male and female rats also showed comparable results, with no significant toxicity effects related to gender differences. These results confirm that AuNPs-DsiRNA does not exert major toxicity effects that can be attributed to gender differences.

#### 3.7.9 Potential of AuNPs-DsiRNA Against Irritation.

**Fig 14:**
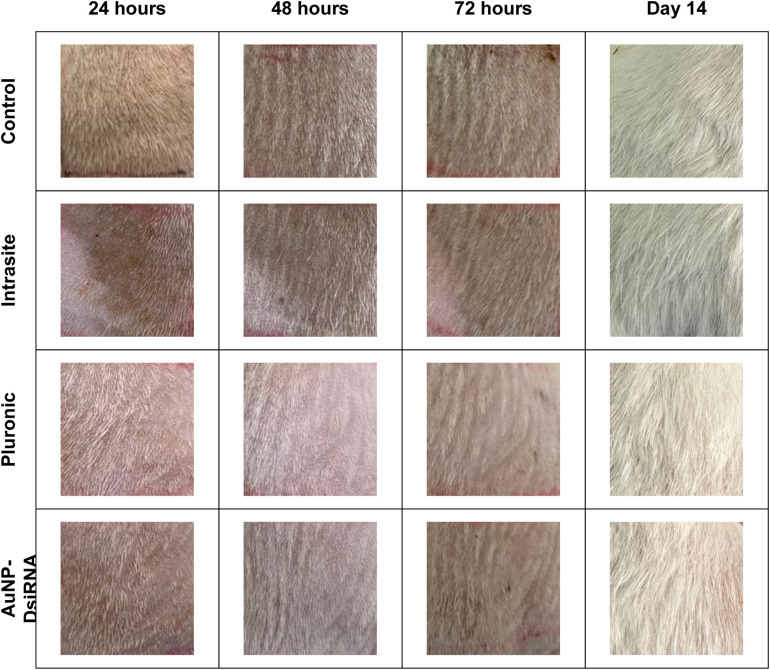
Rat skin after gel application at 24, 48, 72 hours and on day 14, n = 4.

The potential of AuNPs-DsiRNA gel to cause skin irritation was tested, and it was found that it did not produce any unwanted side effects such as redness when applied to the skin, either once or repeatedly (Fig 14). These results were comparable to other groups including the untreated control group.

### 3.8. *In Vivo* efficacy of AuNPs-DsiRNA Gel

#### 3.8.1. Fasting blood glucose levels, mortality, body weight and food intake.

In this study, Wistar rats were divided into two time points, namely 7 and 11 days of treatment, each time with four equal groups. The first group, the untreated diabetic group acted as a control. The second group was a positive control, treated with commercial Intrasite Gel. The third group received Pluronic Gel treatment while the last group received Pluronic AuNPs-DsiRNA gel treatment.

STZ is used to induce a diabetic state in rats due to its properties as a natural antineoplastic methylnitrosourea antibiotic compound, isolated from the bacterium *Streptomyces achromogenes* [88]. STZ is a highly selective pancreatic islet β-cell cytotoxic agent that, when administered in a single high dose, causes complete β-cell necrosis and induces diabetes within 48 hours [22]. The alkylating properties of STZ, similar to cytotoxic nitrosourea compounds, result in β-cell destruction and induce a hyperglycemic state [89].

After induction of diabetes, diabetic animals showed increased food and water intake, frequent urination, defecation, and elevated blood glucose levels compared to pre-STZ conditions. In addition, these diabetic animals experienced weight loss confirming a hyperglycemic and diabetic state [90].

#### 3.8.2 Observations and comparisons.

**Fig 15:**
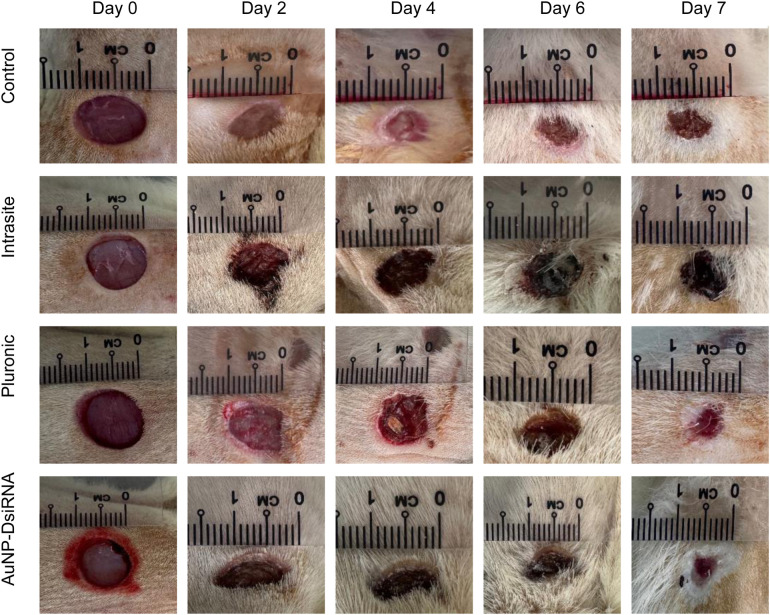
Visual representation of different treatment groups on Days 0, 2, 4, 6, and 7, over the 7-day study period.

**Fig 16:**
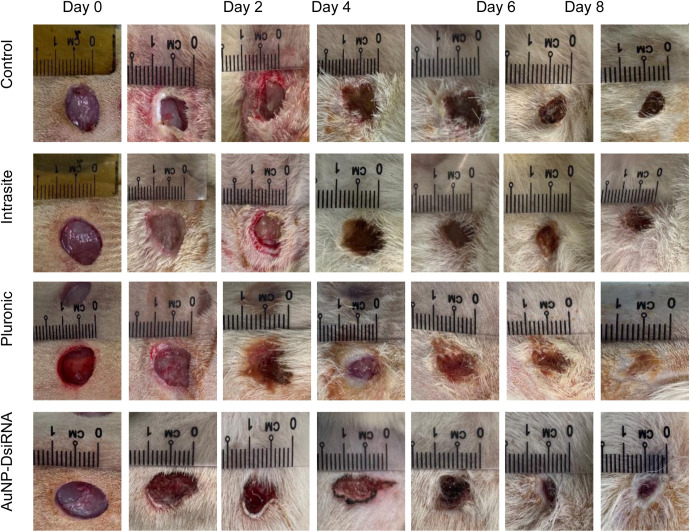
Visual representation of different treatment groups on Days 0, 2, 4, 6, 8, 10, and 11, over the 11-day study period.

In this study, wound measurements were taken every two days for 7 and 11 days before the rats were sacrificed for necropsy. On day 0, all gel treatments were applied to the wound site. By day 2, almost all treatment groups showed signs of infection, including pus at the wound site. However, the AuNPs-DsiRNA treatment group was observed to be the only group with wounds without any signs of pus, while the other groups showed varying degrees of wet wounds and pus formation. This can be seen as shown in Figs 15 and 16.

The antibacterial properties of AuNPs are important because they can prevent infection in diabetic wounds. They work by disrupting the bacterial cell membrane and inhibiting ATP synthesis, effectively reducing bacterial load and preventing the development of infection [91,92].

**Fig 17:**
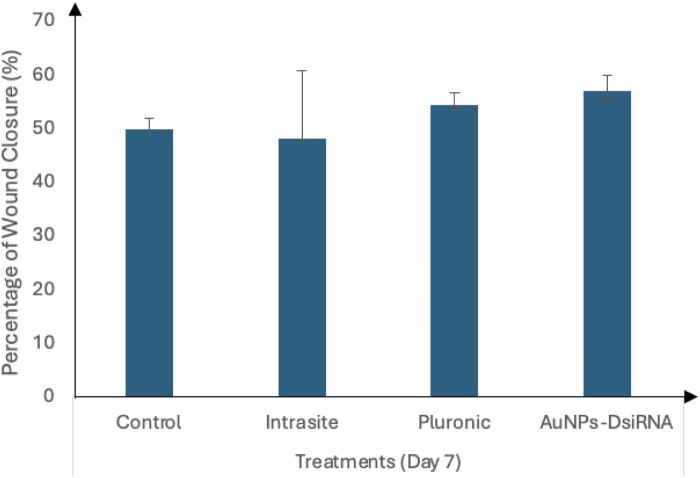
Wound closure percentage for different treatment groups on Day 7 relative to Day 0, n = 4. Footnotes: No significant difference was observed (p > 0.05).

**Fig 18:**
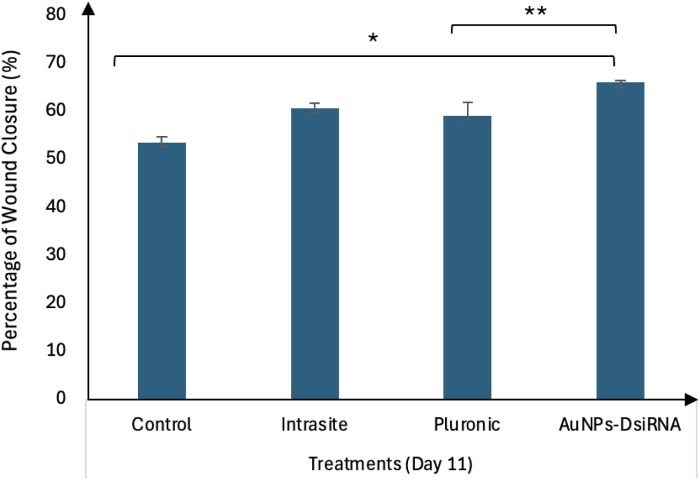
Wound closure percentage for different treatment groups on Day 11 relative to Day 0, n = 4. Footnotes: * – statistically different from the control group (p < 0.05); ** – statistically different from Pluronic (p < 0.05).

At day 7 (Fig 17), the AuNPs-DsiRNA group showed the highest wound closure at 57.19 ± 2.94%, followed by Pluronic (54.54 ± 2.27%), Control (49.9 ± 2.2%), and Intrasite (48.20 ± 12.61%). Meanwhile, on day 11 (Fig 18), the AuNPs-DsiRNA group also maintained the highest wound closure at 66.14 ± 0.35%, followed by Intrasite (60.8 ± 0.93%), Pluronic (59.18 ± 2.75%) and Control (53.58 ± 1.18%). The AuNPs-DsiRNA-treated group showed a statistically significant difference compared to both the untreated control group and the Pluronic group on day 11 (p ≤ 0.05).

AuNPs are dual-functional in that they combine antibacterial and proangiogenic properties, making them effective against multidrug-resistant infections in diabetic wounds. By reducing bacterial growth and enhancing collagen formation and epithelialization, these nanoparticles provide a better healing environment. The combination of the antibacterial properties of AuNPs and the gene silencing effects of DsiRNA in AuNPs-DsiRNA treatment has the potential to promote faster wound closure and stimulate angiogenesis, as evidenced by the formation of new blood vessels [19,20].

Although wound closure can also be observed in some groups, these groups often experience underlying infections, which can pose long-term healing challenges. The AuNPs-DsiRNA group shows that this gel has the potential to help achieve a balanced inflammatory response and provide protection to the wound from external pathogens [91,93].

The AuNPs-DsiRNA gel aids in the early phase of wound healing by accelerating blood clot formation and haemostasis, similar to the natural process of skin healing. When this gel is applied, it interacts with wound elements such as collagen to promote platelet aggregation and blood clot formation. This also promotes the release of chemotactic and growth factors in the wound environment, accelerating the healing process and reducing the risk of infection, especially in diabetic wounds [94]. In chronic wounds, inflammation plays an important role in protecting the wound from invading microbes and preparing the wound for the next stage of healing; however, excessive inflammation can delay healing and compromise wound integrity [20]. Therefore, AuNPs have the potential to modulate the inflammatory response, promoting a balanced environment to aid in the formation of stable tissue and protect underlying tissue.

#### 3.8.3. Gel application and dressing strategy.

Initially, the wound was covered with Tegaderm to maintain moisture. However, this approach may cause problems such as infection in some rats if the wound area becomes too moist, which may attract microbial growth. Alternatively, the treatment gel was applied directly to the wound without a second dressing. This method allowed the gel to remain intact even after two days, resulting in drier wounds and reduced stress on the rats, with no signs of infection throughout the study. In contrast, the group using a semi-occlusive dressing experienced frequent wound infections and complications.

Comparison of dressing techniques showed that the application of the medicated gel was equivalent without the need for additional dressings [95]. This may positively alter the healing process by preventing scab formation and maintaining moisture. Furthermore, the AuNPs-DsiRNA gel was able to maintain a moist environment without the need for frequent dressing changes, which risked promoting bacterial growth as observed in other groups. Therefore, the AuNPs-DsiRNA gel, when used without additional dressings, shows great potential in treating chronic diabetic wounds by maintaining a healing environment free from signs of infection.

AuNPs exhibit broad-spectrum antibacterial activity against common pathogens, including *Staphylococcus aureus* and *Escherichia coli*. Studies have shown that AuNPs can significantly reduce bacterial burden in infected wounds, preventing complications associated with infections such as Methicillin-resistant *Staphylococcus aureus* (MRSA) [96]. The antibacterial mechanism involves disruption of bacterial cell membranes and inhibition of biofilm formation, which is essential for effective wound management [97–99].

#### 3.8.4. Fixation and management in chronic wound care.

Fixation of dressings is important in managing chronic wounds to prevent gel leakage and ensure skin mobility. However, occlusive dressings can cause skin irritation and remove wound tissue during removal, interfering with healing [100].

In diabetic patients, where wound healing is delayed, priority is given to symptom control such as exudate reduction, pain management, and maintaining a sterile environment [101]. Hydrogel dressings help maintain wound moisture, facilitate debridement of necrotic tissue, and allow monitoring without frequent dressing changes [100,102].

In this study, AuNPs-DsiRNA gel was used without additional occlusive dressings and was sufficient to treat diabetic wounds. The gel showed strong antibacterial properties, prevented infection, absorbed exudate well, and maintained an infection free environment. The use of this gel without an occlusive dressing reduces the risk of infection due to trapped moisture, making it an effective solution for chronic wounds, particularly in diabetic patients.

#### 3.8.5. Relative expression of target genes.

The efficacy of wound healing was confirmed by qPCR analysis of PGT and VEGF gene expression using the reference gene GAPDH. Wound tissue samples were collected on days 7 and 11, followed by RNA extraction, cDNA synthesis, and PCR.

**Fig 19:**
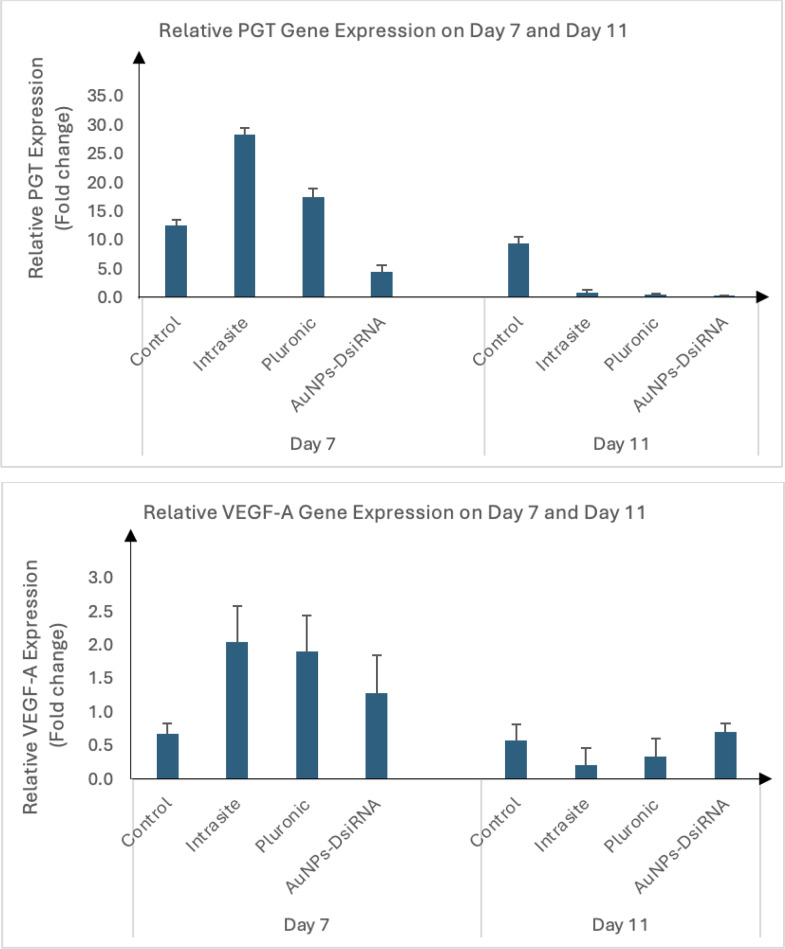
Relative expression of PGT (top) and VEGF-A (bottom), normalized to GAPDH, n = 4.

***3.8.5.1 PGT and Wound Healing.*** Based on Fig 19, PGT expression was found to be high in all groups at day 7, especially in the Intrasite and Pluronic groups. The high PGT expression during the early phase of wound healing may be due to increased prostaglandin activity, which is important for the inflammatory response, vasodilation, and regulation of local immune responses [103,104]. This also indicates a strong early inflammatory response, which is important to prepare the wound for the subsequent healing phase. This response may be closely related to wound tissue formation, indicating haemostasis and active inflammatory activity [105]. Meanwhile, the AuNPs-DsiRNA gel showed lower PGT expression compared to the control group. The absence of infection in the AuNPs-DsiRNA group may contribute to the lower PGT levels due to a more controlled inflammatory response.

At day 11, PGT expression was significantly reduced in all groups, especially AuNPs-DsiRNA gel, followed by Intrasite gel, and Pluronic, as well as the control group. This decrease indicates a transition from the inflammatory phase to the proliferative and remodeling phase of wound healing, where reduction of inflammation is essential for effective tissue repair [103]. Prolonged or excessive PGT expression may impair healing by prolonging chronic inflammation. PGT plays an important role in modulating the effects of prostaglandins, particularly PGE_2_, which are important in angiogenesis and subsequently wound healing. In diabetic conditions, elevated PGT may interfere with PGE_2_ signaling, attenuate the angiogenic response, and prolong inflammation, contributing to chronic wounds [106].

PGT expression of AuNPs-DsiRNA gel decreased to a lesser extent at day 11 compared to day 7, indicating a well-regulated inflammatory response, suitable for healing. The high expression of PGT by the control group may indicate ongoing inflammation, which could potentially delay full healing [105]. This pattern emphasizes the dual role of AuNPs in enhancing initial inflammation for infection control and then modulating the response to prevent chronic inflammation, thereby promoting effective wound healing [20]. These data indicate that the AuNPs-DsiRNA gel can effectively control inflammation, ensuring a robust initial response for wound healing.

***3.8.5.2 VEGF and Wound Healing.*** Based on Fig 19, VEGF expression in the AuNPs-DsiRNA gel group was slightly lower compared to Pluronic and Intrasite gels on day 7. However, on day 11, AuNPs-DsiRNA gel showed the highest VEGF expression over the other gel treatments.

The modest expression of VEGF in the early stages may be due to an inflammatory response focused on infection control. Inflammation during the early phase is characterized by infiltration of neutrophils and macrophages, which are important for new tissue formation in wound healing [107]. As inflammation subsides, the pro-angiogenic properties of AuNPs, combined with the gene silencing effects of DsiRNA, may have increased VEGF expression by day 11.

The potent antibacterial effect of the AuNPs-DsiRNA gel may have provided a better wound environment over time, free from infection. This clean environment enhances the subsequent phases of wound healing, including angiogenesis, which is important for increased VEGF expression [108]. By day 11, the cumulative effects of the gel components may have further enhanced tissue regeneration. The presence of a tissue layer at the wound site and the absence of infection support the transition from the inflammatory to the proliferative phase, where increased VEGF activity is essential for new blood vessel formation [109].

Initially, the AuNPs-DsiRNA gel focused on wound stabilization and infection prevention, which may have delayed the increase in VEGF. However, the subsequent shift towards active healing resulted in a significant increase in VEGF by day 11, supporting tissue repair and enhanced blood vessel formation [110]. The combination of AuNPs and DsiRNA exerted a time-dependent effect, with a focus on gene silencing and microbial control at an early stage, followed by a synergistic increase in VEGF expression, aiding wound closure and healing.

## 4. Conclusion

This study highlights the potential of a thermoresponsive AuNPs-DsiRNA gel as an innovative treatment for diabetic wounds. The upscaled synthesis of AuNPs, optimized via RSM, achieved a 100-fold yield increase while maintaining excellent stability (>+30 mV) and a narrow size distribution. The incorporation of DsiRNA resulted in a formulation with potent antimicrobial activity against *P. aeruginosa* and *S. aureus*, high entrapment efficiency (>70%), good binding efficacy, and sustained release over 8 hours, ensuring prolonged wound interaction. Safety assessments, including sub-acute toxicity and skin irritation studies, confirmed the gel’s biocompatibility, with no adverse effects detected in haematological, biochemical, or histopathological evaluations.

The gel also showed superior wound healing efficacy in vivo, with faster wound closure, absence of infection, and effective modulation of key genes. The downregulation of PGT and upregulation of VEGF enhanced angiogenesis and reduced inflammation, critical factors for diabetic wound healing. Overall, the upscaled thermoresponsive AuNPs-DsiRNA gel demonstrated significant therapeutic potential, combining safety, antimicrobial properties, and enhanced healing, positioning it as a promising solution for diabetic wound care.

## Supporting information

S1 File Tables S1–S8, including the experimental independent variables and coded levels used in the CCD design (Table S1), rheological characterization data of the thermoresponsive gel (Table S2), drug release profiles of AuNPs and DsiRNA from the thermoresponsive gel (Tables S3–S4), relative expression of target genes PGT and VEGF-A on days 7 and 11 determined by qPCR (Tables S5–S6), and wound observations and comparisons following 7 and 11 days of treatment (Tables S7–S8). (PDF)
