# Supplementary material for: Upscaling, toxicity and efficacy of multifaceted dressing embedded with dsirna-loaded gold nanoparticles for enhancing diabetic wound treatment
Source: PLoS One. 2025 Sep 5;20(9):e0327375. doi: 10.1371/journal.pone.0327375 (PMC12412931; doi:10.1371/journal.pone.0327375)
Supplement: S1 File — (PDF) [file pone.0327375.s001.pdf]

Supporting Information (Supplementary Data)

S1 Table. Experimental Independent Variables and Their Coded Levels Used in the Central Composite Design (CCD)

This table outlines the experimental factors and their respective coded levels applied in the CCD approach for optimizing gold nanoparticle (AuNP) synthesis.

Independent variables	units	Low (-1)	High (+1)	-alpha	+alpha
HauCl4 conc.	(mL)	0.6	0.8	0.558579	0.841421
TMM conc.	(mL)	3.2	5.5	2.72365	5.97635

S2 Table. Rheological Characterization Data of the Thermoresponsive Gel

This table presents the raw data used to generate the rheology graphs, including viscosity and shear rate measurements for assessing the non-Newtonian flow behavior of the gel formulation.

Shear Rate [1/s]	Gel 1	Gel 2	Gel 3	Gel 4
0.1	137.45	180.41	228.99	148.88
0.121	205.865	251.305	260.485	207.435
0.153	267.54	333.905	288.54	281.145
0.195	280.02	346.235	285.215	295.765
0.247	261.29	325.86	268.045	281.08
0.313	253.005	309	255.49	265.67
0.397	242.83	295.6	247.83	256.045
0.504	236.085	286.235	243.535	247.74
0.64	231.345	282.875	242.46	243.655
0.811	231.54	283.365	243.83	242.285
1.03	233.125	286.23	246.99	243.98
1.31	236.92	290.485	250.975	247.3
1.66	240.2	295.445	255.165	251.5
2.1	240.62	301.72	259.385	255.91
2.67	246.8	307.06	263.975	261.535
3.38	249.25	313.695	269.29	267.33
4.29	253.59	319.92	274.81	272.585
5.44	258.13	325.665	280.435	276.37
6.91	262.69	331.22	286.52	280.11
8.76	265.66	336.08	290.145	284.5
11.1	271.09	342.175	292.8	288.885
14.1	275.52	346.52	297.035	293.29
17.9	278.075	352.665	302.75	299.21
22.7	282.4	359.16	309.815	306.435

28.8	290.25	365.455	318.5	311.945
36.5	300.78	376.895	328.935	322.035
46.3	313.06	387.71	339.15	331.09
58.8	322.105	400.965	351.43	344.125
74.5	331.66	415.815	363.93	356.725
94.6	343.845	430.58	376.07	369.47
120	356.13	448.245	392.23	384.45
152	369.83	467.38	408.42	400.255
193	385.89	489.31	427.05	419.625
245	403.925	512.355	446.93	438.81
311	423.27	538.175	469.325	460.43
394	444.735	565.955	493.275	484.125
500	468.405	594.44	519.115	509.61
500	469.37	595.32	519.715	509.84
413	451.27	572.295	499.195	490.31
326	430.145	545.32	475.07	467.69
257	409.99	519.355	452.96	446.795
202	392.655	495.335	432.415	427.39
160	377.03	472.765	414.18	408.655
126	362.2	452.55	396.585	392.135
99.2	348.585	434.7	380.725	377.56
78.2	335.395	416.755	365.81	362.84
61.6	325.535	401.67	352.405	350.06
48.6	315.25	388.045	340.685	338.595
38.3	304.32	375.12	328.91	326.685
30.2	296.205	363.295	318.065	317.1
23.8	284.4	352.3	308.34	306.67
18.8	280.45	343.315	299.64	298.7
14.8	270.925	334.705	291.76	290.955
11.7	265.81	326.82	284.94	283.84
9.19	257.365	320.145	279.485	277.455
7.24	256.355	315.055	275.59	272.61
5.71	251.56	311.175	272.83	268.285
4.5	248.305	307.83	270.04	264.735
3.55	240.9	304.785	267.11	261.47
2.8	241.83	301.95	264.02	258.485
2.2	238.28	298.575	260.42	255.385
1.74	235.91	295.065	256.695	252.13
1.37	233.85	291.18	252.59	248.75
1.08	227.675	287.325	248.485	245.135
0.851	225.17	283.16	244.46	241.47
0.671	221.335	278.8	240.34	237.93
0.529	217.91	274.75	236.38	234.19
0.417	215.13	270.495	232.26	230.635

0.329	211.935	266.48	227.725	227.19
0.259	208.125	262.2	223.28	223.58
0.204	205.74	258.095	218.905	220.24
0.161	201.64	253.765	214.67	216.745
0.127	200.18	249.95	210.96	213.605
0.1	195.385	245.99	207.35	210.32

S3 Table. Drug Release Profile Data of AuNPs from Thermoresponsive Gel
This table provides the cumulative drug release data of gold nanoparticles (AuNPs) from the thermoresponsive gel formulation over time, measured via UV-Vis spectrophotometry.

Mean (nm)	Concentration	Time (Hr)	Amount released (mg)	Cumulative release (mg)	Cumulative release (%)	SD
0.136	0.02804121	1	0.028	0.028	1.122	0.526
0.411	0.18902939	2	0.189	0.217	8.683	0.116
0.113	0.01457675	3	0.015	0.232	9.266	0.018
0.159	0.04150568	4	0.042	0.273	10.926	0.133
0.212	0.07253249	5	0.073	0.346	13.827	0.040
0.576	0.28562229	6	0.286	0.631	25.252	0.136
0.64	0.32308863	7	0.323	0.954	38.176	0.152
0.223	0.07897202	8	0.079	1.033	41.335	0.010
0.314	0.13224447	24	0.132	1.166	46.625	0.043
0.236	0.08658237	48	0.087	1.252	50.088	0.012

S4 Table. Drug Release Profile Data of DsiRNA from Thermoresponsive Gel
This table provides the cumulative drug release data of gold nanoparticles (DsiRNA) from the thermoresponsive gel formulation over time, measured via UV-Vis spectrophotometry.

Mean (nm)	Concentration	Time (Hr)	Amount released (mg)	Cumulative release (mg)	Cumulative release (%)	SD
0.184	0.05614097	1	0.056	0.056	2.246	0.035
0.133	0.02628498	2	0.026	0.082	3.297	0.226
0.163	0.04384732	3	0.044	0.126	5.051	0.011
0.249	0.09419272	4	0.094	0.220	8.819	0.008
0.331	0.14219646	5	0.142	0.363	14.506	0.004
0.709	0.36348203	6	0.363	0.726	29.046	0.254
0.316	0.13341529	7	0.133	0.860	34.382	0.295
0.342	0.14863599	8	0.149	1.008	40.328	0.033
0.586	0.29147641	24	0.291	1.300	51.987	0.109
0.339	0.14687976	48	0.147	1.447	57.862	0.017

S5 Table. Relative Expression of Target Genes (qPCR Data) for PGT on Days 7 and 11 – Average Values

This table presents the average relative expression levels of PGT on days 7 and 11 as determined by qPCR analysis.

Day	Group	Value	SD
7	Control	12.5	0.97
	Intrasite	28.4	1.13
	Pluronic	17.5	1.44
	AuNPs-DsiRNA	4.5	1.23
11	Control	9.4	1.22
	Intrasite	0.8	0.5
	Pluronic	0.5	0.23
	AuNPs-DsiRNA	0.3	0.05

S6 Table. Relative Expression of Target Genes (qPCR Data) for VEGF-A on Days 7 and 11 – Average Values

This table presents the average relative expression levels of VEGF-A on days 7 and 11 as determined by qPCR analysis.

Day	Group	Value	SD
7	Control	0.7	0.16
	Intrasite	2	0.53
	Pluronic	1.9	0.54
	AuNPs-DsiRNA	1.3	0.56
11	Control	0.6	0.24
	Intrasite	0.2	0.26
	Pluronic	0.3	0.27
	AuNPs-DsiRNA	0.7	0.12

S7 Table. Wound Observations and Comparisons Based on Image and Chart Data Following 7 Days of Treatment

This table presents qualitative and quantitative assessments of wound progression after 7 days of treatment across all experimental groups.

Day	Group	Mean	Std. Deviation, SD
**Day 2**	Control	17.21	1.49523
	Intrasite	21	11.21453
	Pluronic	23.1175	5.0934
	AuNPs-DsiRNA	15.915	4.64034
**Day 4**	Control	31.9375	1.71107
	Intrasite	26.6933	11.77983
	Pluronic	34.4633	5.99032
	AuNPs-DsiRNA	24.86	4.17338
**Day 6**	Control	41.8225	2.37565
	Intrasite	34.5333	11.11426
	Pluronic	48.51	3.91501
	AuNPs-DsiRNA	40.8875	3.1901
**Day 7**	Control	49.895	2.20664
	Intrasite	48.2033	12.61578
	Pluronic	54.5425	2.27036
	AuNPs-DsiRNA	57.1875	2.94234

S8 Table. Wound Observations and Comparisons Based on Image and Chart Data Following 11 Days of Treatment

This table presents qualitative and quantitative assessments of wound progression after 11 days of treatment across all experimental groups.

Day	Group	Mean	Std. Deviation, SD
**Day 2**	Control	23.1575	3.48228
	Intrasite	20.865	5.08129
	Pluronic	32.3025	4.50641
	AuNPs-DsiRNA	30.36	2.68784
**Day 4**	Control	32.6175	3.18455
	Intrasite	27.895	4.14965
	Pluronic	42.0725	6.13431
	AuNPs-DsiRNA	38.2125	2.03245
**Day 6**	Control	40.795	1.12846
	Intrasite	32.8925	4.21113
	Pluronic	44.845	6.77185
	AuNPs-DsiRNA	43.28	2.19431
**Day 8**	Control	44.9925	1.21049
	Intrasite	43.7825	3.74883
	Pluronic	48.815	3.92417
	AuNPs-DsiRNA	48.49	2.28839
**Day 10**	Control	48.6575	0.53756
	Intrasite	53.245	1.26012
	Pluronic	53.8125	3.79043
	AuNPs-DsiRNA	54.8075	1.84664
**Day 11**	Control	53.5775	1.17267
	Intrasite	60.8	0.92791
	Pluronic	59.175	2.75049
	AuNPs-DsiRNA	66.1375	0.35901
